# Global, regional, and national mortality trends of female breast cancer by risk factor, 1990–2017

**DOI:** 10.1186/s12885-021-08217-5

**Published:** 2021-04-24

**Authors:** Hui Liu, Wenjie Shi, Zhi Jin, Rui Zhuo, Jie Dong, Qiufeng Lao, Shengle Li, Weiyi Pang

**Affiliations:** 1 School of Public Health, Guilin Medical University, 1 Zhiyuan Road, Guilin, Guangxi 541199 P.R. China; 2 University Hospital for Gynecology, Pius-Hospital, University Medicine Oldenburg, 26121 Oldenburg, Germany; 3 Department of Neurology, Shanghai Fifth People’s Hospital Fudan University, Shanghai, 200240 P.R. China; 4 Department of Breast Surgery, Guilin TCM Hospital of China, Affiliated to Guang Xi University of Chinese Medicine, Guilin, Guangxi 541000 P.R. China

**Keywords:** Female breast cancer, Mortality, Alcohol, Obesity, Diabetes, Smoking

## Abstract

**Background:**

Female breast cancer (FBC) is a malignancy involving multiple risk factors and has imposed heavy disease burden on women. We aim to analyze the secular trends of mortality rate of FBC according to its major risk factors.

**Methods:**

Death data of FBC at the global, regional, and national levels were retrieved from the online database of Global Burden of Disease study 2017. Deaths of FBC attributable to alcohol use, high body-mass index (BMI), high fasting plasma glucose (FPG), low physical activity, and tobacco were collected. Estimated average percentage change (EAPC) was used to quantify the temporal trends of age-standardized mortality rate (ASMR) of FBC in 1990–2017.

**Results:**

Worldwide, the number of deaths from FBC increased from 344.9 thousand in 1990 to 600.7 thousand in 2017. The ASMR of FBC decreased by 0.59% (95% CI, 0.52, 0.66%) per year during the study period. This decrease was largely driven by the reduction in alcohol use- and tobacco-related FBC, of which the ASMR was decreased by 1.73 and 1.77% per year, respectively. In contrast, the ASMR of FBC attributable to high BMI and high FPG was increased by 1.26% (95% CI, 1.22, 1.30%) and 0.26% (95% CI, 0.23, 0.30%) per year between 1990 and 2017, respectively.

**Conclusions:**

The mortality rate of FBC experienced a reduction over the last three decades, which was partly owing to the effective control for alcohol and tobacco use. However, more potent and tailored prevention strategies for obesity and diabetes are urgently warranted.

**Supplementary Information:**

The online version contains supplementary material available at 10.1186/s12885-021-08217-5.

## Introduction

Female breast cancer (FBC) is the most frequently diagnosed carcinoma in women and is also the leading cause of cancer death in over 100 countries [1]. Worldwide, a total of 0.63 million deaths were attributed to FBC in 2018 [1]. Although a favorable trend in FBC mortality rate has been witnessed in recent years, particularly in developed countries, FBC still constitutes a major public health concern owing to the increasing reservoir of incident cases [2–4]. The risk factors of FBC, both environmental and genetic, have been extensively investigated in previous studies [5–7]. For instance, family history of breast cancer or ovarian cancer and inherited mutations in *BRCA1* and *BRCA2* accounts for 5 to 10% of FBC cases [4]. The remainder are might largely ascribed to environmental risk factors, including alcohol drinking, smoking, physical inactivity, and obesity [8–11]. Moreover, psychological and social risk factors, such as chronic stress, dementia, and family disintegrity etc., also play roles in the development of FBC [12, 13]. The patterns of incidence and mortality of FBC are therefore varied across the world due to the geographical heterogeneities in these risk factors [4]. For example, in the USA, the recent trend of FBC incidence was mainly driven by the changes in body mass index (BMI) [5]. Whereas patterns of breast cancer risk in Chinese women are only partly consistent with the well-determined risk factors in women in high-income countries [14, 15]. Understanding the risk pattern of FBC is instrumental to establish more tailored prevention strategies in each country.

However, FBC is deemed as a multi-etiological disease and it is hard to disentangle the contribution of single risk factor. Fortunately, based on comprehensive surveillance cancer data and advanced mathematical models, the Global Burden of Disease (GBD) study provides information on contributions of several well-defined risk factors to FBC. The GBD data have been widely used to learn the burden of diseases, including cancers [16–18]. Different to previous studies [4, 18], herein, we use the data from GBD database to analyze the temporal trends of FBC mortality rates by risk factor at the global, regional, and national levels. Our results are not only the important complement to the previous studies, but provide more insight into the FBC prevention.

## Materials and methods

We collected the data of FBC mortality attributable to all causes, high BMI, alcohol use, high fasting plasma glucose, low physical activity, and smoking from 1990 to 2017 using the online Global Health Data Exchange (GHDx) query tool [19]. The mortality data were stratified by calendar year, risk factor, and countries or territories. These countries or territories were categorized into 5 regions in terms of socio-demographic index (SDI) and 21 regions in terms of geography (e.g., East Asia). The general methods of the GBD study and the methods for the estimations of disease burden caused by 84 behavioral, environmental and occupational, and metabolic risks or clusters of risks have been detailed in previous studies [20]. As summarized by Zhang et al. [21], the Global Burden of Diseases, Injuries, and Risk Factors Study comparative risk assessment (CRA) is a comprehensive and comparable approach to risk factor quantification that offers a useful tool for synthesizing evidence on risks and risk–outcome associations. The relative risk by level of exposure, or by cause, for mortality or morbidity can be found in previously published and unpublished studies or in secondary studies that summarize relative risks. For FBC, mortality from 10 risk factors at different levels were calculated, including “all risk factors” at level 0, metabolic risks and behavioral risks at level 1, tobacco, alcohol use, high fasting plasma glucose (FPG), low physical activity, and high BMI at level 2, and smoking and secondhand smoke at level 3. In this study, we retrieved the FBC mortality data on 5 risk factors at level 2. The details of methods for calculating FBC mortality from these 5 risk factors were shown in Supplement.

We used the estimated average percentage change (EAPC) to measure the temporal trends of FBC age-standardized mortality rate (ASMR) between 1990 and 2017. Briefly, the EAPC is a summative and widely used index to quantify the secular trends in a specified interval. The calculation of EAPC was borrowed from previous studies [4, 21]. We fitted linear regression between ASMR and calendar year as: *y = α + βx + ɛ,* where *y =* ln (ASMR) and *x* = calendar year. The EAPC was calculated as 100 × (*exp*(*β*)-1), and its 95% confidence interval (CI) can also obtain from this model. All statistical tests were implemented in the R program (version 3.6.3). A two-side tested *P* value < 0.05 was considered statistically significant.

## Results

### Trends of FBC mortality by region

**Fig. 1:**
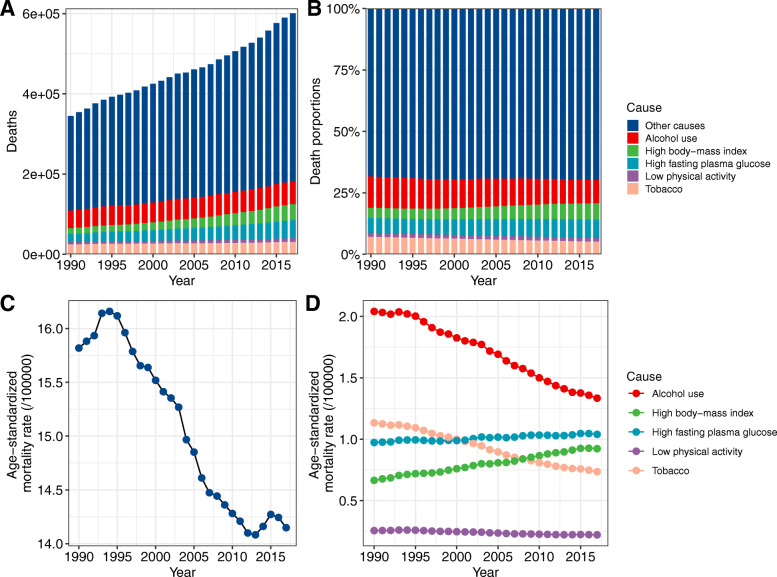
The temporal trends of death number and mortality rate of female breast cancer (FBC) at the global level. **a**, the increasing trend of death number of FBC; **b**, the proportions of FBC related deaths attributable to different risk factors; **c**, the decreasing trend of age-standardized mortality rate of FBC; **d**, the temporal trends of FBC attributable to different risk factors)

**Fig. 2:**
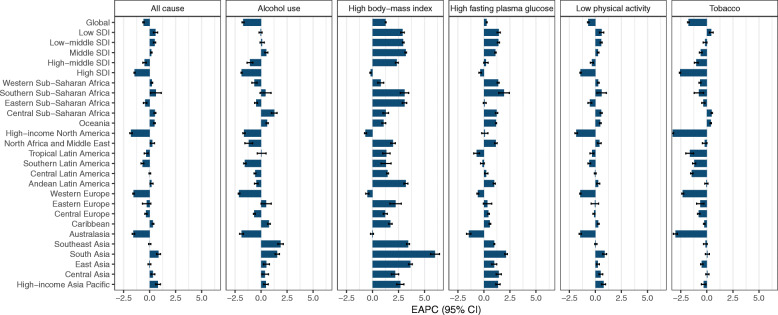
The estimated average percentage change (EAPC) of mortality rate of female breast cancer (FBC) at the global and regional level by risk factor

**Fig. 3:**
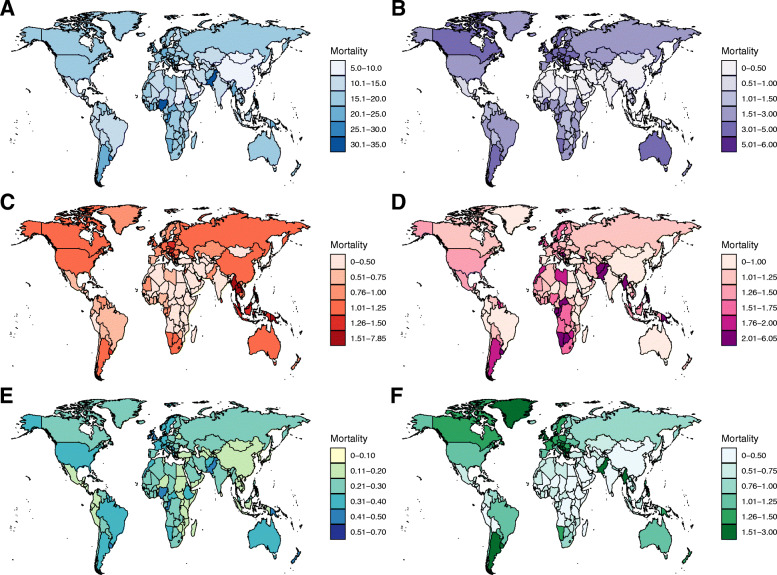
The global distribution of age-standardized mortality rate of female breast cancer (FBC) by risk factor (**a** to **f** displays the mortality of FBC attributable to all causes, alcohol use, high BMI, high fasting plasma glucose, low physical activity, and tobacco, respectively)

**Fig. 4:**
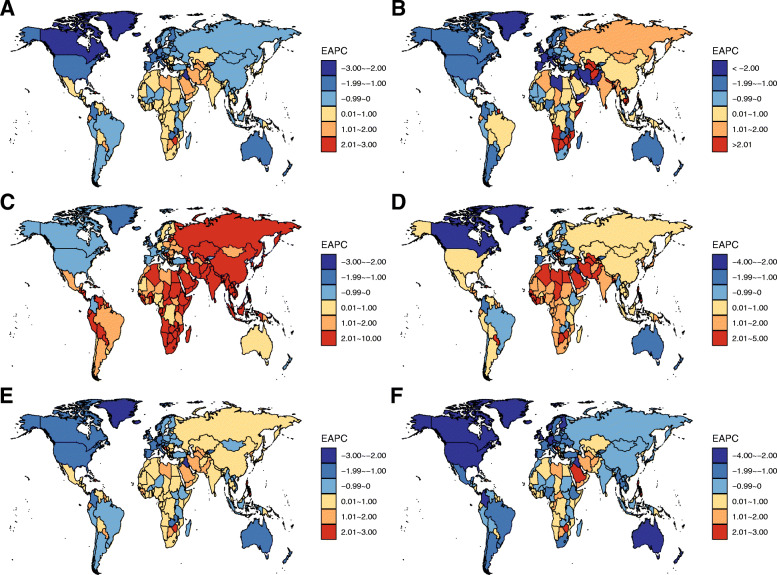
The estimated average percentage change (EAPC) of mortality rate of female breast cancer (FBC) at the national level by risk factor (**a** to **f** displays the EAPC of FBC attributable to all causes, alcohol use, high BMI, high fasting plasma glucose, low physical activity, and tobacco, respectively)

Worldwide, the number of deaths from FBC increased from 344.9 thousand in 1990 to 600.7 thousand in 2017. Whereas the ASMR of FBC decreased by 0.59% (95% CI, 0.52, 0.66%) per year during the study period (Table 1; Fig. 1). The highest ASMR was found in regions with high or middle-high SDIs, in which the ASMR experienced a pronounced decrease from 1990 to 2017 (Table 1; Fig. 2). In contrast, the ASMRs in regions with low to middle SDIs were significantly increased with different magnitudes. At the GBD regional level, the higher ASMR of FBC was mostly observed in regions with advanced economies, including Western Europe, high-income North America, and Australasia, in which the ASMR had experienced a significant decrease (Table 1; Fig. 2). In Sub-Saharan Africa, the ASMR of FBC was exceeded the global average and showed an increasing trend during the last three decades (Table 1; Fig. 2). At the national level, in 2017, the highest ASMR was found in Pakistan (34.14/100,000), followed by Tonga, the Bahamas, and Nigeria (Fig. 3a). Between 1990 and 2017, we found that 97 countries or territories, most were located in Africa and South Asia, experienced a significant increase in ASMR of FBC. The greatest increase was found in Zimbabwe (EAPC = 2.64, 95% CI, 1.80, 3.49), followed by Mauritius and Lesotho (Table S1; Fig. 4a). In contrast, a total of 70 countries or territories, most were located in Europe and the Americas, experienced a significant decrease in ASMR of FBC. The greatest decrease was found in Bermuda (EAPC = − 2.68, 95% CI, − 2.87, − 2.49), followed by Iraq and Greenland (Table S1; Fig. 4a).

**Table 1:** The death number and age-standardized mortality rate of female breast cancer between 1990 and 2017, by region and risk factors

	1990		2017		1990–2017
	Deaths (× 1000)	ASMR (/10^5^)	Deaths (× 1000)	ASMR (/10^5^)	EAPC (95%CI)
Global	344.9	15.82	600.7	14.15	−0.59 (− 0.66,-0.52)
Socio-demographic index					
High	153.7	21.85	178.5	15.37	−1.46 (− 1.51, − 1.40)
High-middle	70.6	13.55	121.2	12.58	−0.49 (−0.65, − 0.33)
Middle	57.4	10.95	139.4	11.83	0.16 (0.09, 0.22)
Low-middle	41.7	13.65	106.3	16.30	0.48 (0.37, 0.58)
Low	20.5	11.53	58.6	15.34	0.57 (0.37, 0.77)
GBD region					
High-income Asia Pacific	8.6	7.65	18.4	8.99	0.80 (0.57, 1.03)
Central Asia	3.6	12.69	6.1	13.86	0.32 (0.14, 0.50)
East Asia	42.0	8.58	90.6	8.58	−0.03 (−0.16, 0.11)
South Asia	34.5	11.07	106.9	15.02	0.85 (0.67, 1.03)
Southeast Asia	22.7	14.64	50.2	14.93	0.01 (−0.09, 0.12)
Australasia	3.0	24.16	4.1	16.72	−1.57 (−1.68, −1.45)
Caribbean	2.4	17.08	5.1	18.78	0.32 (0.25, 0.40)
Central Europe	15.7	19.01	20.2	17.52	−0.36 (−0.46, −0.25)
Eastern Europe	26.1	15.48	32.2	16.05	−0.29 (− 0.70, 0.12)
Western Europe	84.2	26.34	87.4	18.07	−1.55 (−1.61, − 1.49)
Andean Latin America	1.2	10.53	3.2	11.32	0.18 (0.04, 0.33)
Central Latin America	5.6	11.80	15.5	12.20	0.01 (−0.06, 0.09)
Southern Latin America	5.9	22.77	8.9	19.64	−0.69 (− 0.82, − 0.55)
Tropical Latin America	7.9	15.22	18.9	14.65	−0.34 (− 0.53, − 0.14)
North Africa and Middle East	10.5	11.00	27.3	11.79	0.25 (0.06, 0.45)
High-income North America	49.1	25.31	54.8	16.99	−1.79 (−1.91, −1.68)
Oceania	0.3	21.22	0.8	23.19	0.46 (0.38, 0.53)
Central Sub-Saharan Africa	1.9	14.04	5.0	16.49	0.51 (0.43, 0.60)
Eastern Sub-Saharan Africa	7.0	16.25	14.5	15.69	−0.43 (−0.60, −0.27)
Southern Sub-Saharan Africa	2.4	14.61	5.4	16.52	0.60 (0.08, 1.12)
Western Sub-Saharan Africa	10.2	22.02	25.1	23.87	0.21 (0.13, 0.28)
Risk factors					
Alcohol use	44.2	2.04	56.8	1.33	−1.73 (−1.82, −1.63)
High BMI	14.1	0.66	40.0	0.92	1.26 (1.22, 1.30)
High FPG	20.8	0.97	44.5	1.04	0.26 (0.23, 0.30)
Low physical activity	5.5	0.26	9.4	0.22	−0.69 (−0.76, −0.63)
Tobacco	24.9	1.13	31.3	0.74	−1.77 (−1.85, −1.70)

### Trends of FBC mortality attributable to alcohol use

**Fig. 5:**
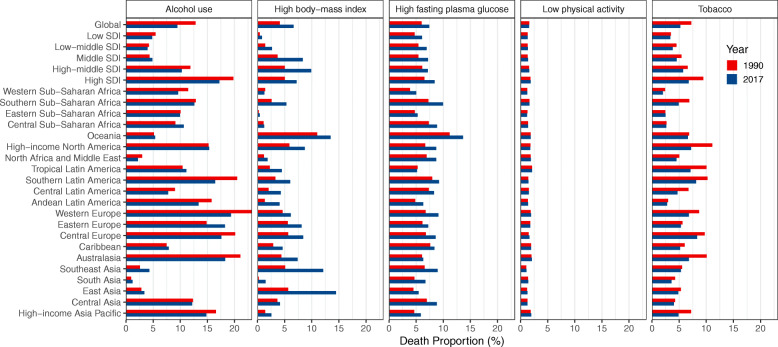
The proportions of female breast cancer related deaths attributable to different risk factors at the global and regional level

While the number of FBC deaths attributable to alcohol use increased from 44.2 thousand in 1990 to 56.8 thousand in 2017, the corresponding ASMR was decreased from 2.04/100,000 to 1.33/100,000 in this period (Table 1; Fig. 1). Globally, alcohol use accounted for nearly 10% of total deaths from FBC in 2017 (Fig. 1). This proportion was high in Western Europe, Australasia, and Southern Latin America (Fig. 5). In these regions, the ASMRs of alcohol use related-FBC were significantly decreased. The greatest decrease was found in high-income North America (Fig. 2; Table S1). In most developing regions, including South Asia, Central Asia, and Caribbean, this rate was significantly increased with different magnitudes (Fig. 2; Table S1). At the national level, the ASMR of FBC attributable to alcohol use was high in most European countries, including Denmark (5.60/100,000), Serbia (5.00/100,000), and Luxembourg (4.90/100,000) (Fig. 3b). A total of 86 and 80 countries or territories experienced a significant increase and decrease in the ASMR, respectively (Fig. 4b; Table S1). The greatest increase was found in Namibia (EAPC = 7.13, 95% CI, 5.28, 9.01), followed by Sri Lanka and Vietnam. Nearly all countries located in Europe and North America were experienced a significant decrease (Fig. 4b; Table S1).

### Trends of FBC mortality attributable to high BMI

In 2017, high BMI accounted for approximately 6.7% of total deaths (40.0 thousand) from FBC (Table 1; Figs. 1 & 5). Between 1990 and 2017, the ASMR of FBC attributable to high BMI was increased by 1.26% (95% CI, 1.22, 1.30%) per year. This increasing trend was also seen in most regions (Figs. 2). At the regional level, the most pronounced increase was observed in South Asia (EAPC = 6.00, 95% CI, 5.56, 6.44), followed by East Asia and Southeast Asia. At the national level, the highest ASMR of high BMI related-FBC was found in Fiji (7.85/100,000), followed by Tonga and American Samoa (Fig. 3c). More than 80% of total countries (160/195) were experienced a significant increase during the study period. The greatest increase was found in Bangladesh (EAPC = 9.41, 95% CI, 8.65, 10.18), followed by India and Equatorial Guinea (Fig. 4c; Table S1). On the contrary, only 19 countries experienced a remarkable decrease in ASMR of high BMI related-FBC. The greatest decrease was found in Bermuda (EAPC = − 2.27, 95% CI, − 2.62, − 1.92), followed by Switzerland and the UK (Fig. 4c; Table S1).

### Trends of FBC mortality attributable to high FPG

From 1990 to 2017, the death number of FBC attributable to high FPG was doubled from 20.8 thousand to 44.5 thousand, which accounted for 7.5% of total deaths from FBC in 2017 (Table 1). The corresponding ASMR was increased from 0.66/100,000 to 0.92/100,000 (Table 1; Fig. 1). The temporal trends of ASMR showed different patterns across the regions, even in adjacent regions (Fig. 2). For example, the ASMR was decreased in Western Europe, whereas increased in Central Europe. At the national level, the highest ASMR was found in Fiji (6.04/100,000), followed by Tonga and American Samoa in 2017 (Fig. 3d). A significant increase in the ASMR was witnessed in 139 countries, most were not advanced in economy (Fig. 4d). The greatest increase was found in Mauritius (EAPC = 4.80, 95% CI, 4.39, 5.22), followed by Zimbabwe and Philippines (Table S1). In contrast, we found that a total of 42 countries were experienced a significant decrease in the ASMR during the study period, most were located in Europe (Fig. 4d). The greatest decrease was found in Greenland (EAPC = − 3.11, 95% CI, − 3.31, − 2.90), followed by Canada and Bermuda (Table S1).

### Trends of FBC mortality attributable to low physical activity

In 2017, 9.4 thousand deaths were caused by low physical activity related-FBC. The corresponding ASMR was decreased by 0.69% per year between 1990 and 2017 (Table 1; Fig. 1). This decreasing trend was mainly driven by the decrease in developed regions, including high-income North America, Western Europe, and Australasia (Fig. 2). However, in developing regions such as Asia and Sub-Saharan Africa, the ASMR experienced a significant increase. At the national level, the highest ASMR was observed in Tonga (0.69/100,000), followed by the Bahamas and Fiji in 2017 (Fig. 3e). Between 1990 and 2017, a total of 107 countries, most were located in Asia and Africa, were experienced a significant increase in the ASMR. The greatest increase was found in Mauritius (EAPC = 2.67, 95% CI, 2.42, 2.93), followed by Zimbabwe and Lesotho (Fig. 4e; Table S1). In the same period, 68 countries experienced a significant decrease in the ASMR. The most pronounced decrease was seen in Bermuda (EAPC = − 2.56, 95% CI, − 2.76, − 2.37), followed by Iraq and Greenland (Fig. 4e; Table S1).

### Trends of FBC mortality attributable to tobacco use

Globally, the death number of FBC attributable to tobacco use was increased from 24.9 thousand in 1990 to 31.3 thousand in 2017, which accounted for 5.2% of total FBC-related deaths (Table 1). The corresponding ASMR was decreased by 1.77% per year during the study period (Table 1; Fig. 1). This decreasing trend was observed in most regions, with the greatest decrease was found in high-income North America (EAPC = − 3.29, 95% CI, − 3.39, − 3.20) (Fig. 2). At the national level, the higher ASMR was mostly observed in European countries (Fig. 3f). Serbia had the highest ASMR (2.65/100,000) in 2017, followed by Lebanon and Montenegro. A total of 78 countries, most were located in Africa and Middle East, experienced a significant increase in the ASMR from 1990 to 2017. The greatest increase was found in Zimbabwe (EAPC = 2.99, 95% CI, 2.12, 3.85), followed by Lesotho and Saudi Arabia (Fig. 4f; Table S1). In contrast, 92 countries experienced a significant decrease in the ASMR. The greatest decrease was found in the UK (EAPC = − 3.60, 95% CI, − 3.66, − 3.54), followed by Iceland and Canada (Fig. 4f; Table S1).

## Discussion

In this study, we report the temporal trends of AMSR of FBC attributable to all causes and five well-determined risk factors. Globally, although the overall ASMR of FBC was decreased over the last three decades, ASMR of FBC attributable to high BMI and high FPG was significantly increased. The decrease in FBC mortality rate was at least partly driven by the effective control for alcohol and tobacco use. The improvement in clinical treatment and management of FBC cases also serves as contributors for this decrease [22]. Additionally, the changing trends of the ASMR by risk factors are varied across the world. For example, the ASMR was decreased in most developed countries, irrespective of risk factors. However, in most developing countries, this rate was seen a significant increase. Our findings highlight that FBC is still a major public health concern and suggest that overweight (or obesity) and diabetes have become the major drivers for FBC.

FBC is a complex disease involving both genetic and environmental factors. Genetically, mutations in *BRAC1* and *BRAC2* have long been determined for association with FBC carcinogenesis [23]. Whereas evidences from epidemiological studies revealed that these mutations are associated with at most one-third as many breast cancer cases in the general population [24]. The remainder of FBC cases might be ascribed to environmental risk factors. Previous migrant epidemiological studies also suggested that environmental factors, including individual behaviors and living conditions, might play a more important role in FBC development than genetic factors [25, 26]. From this point of view, unveiling the major environmental risk factors in a population is more informative for cancer prevention.

For FBC, as shown in this study, the main risk factors including alcohol use, smoking, overweight and obesity, diabetes, and physical inactivity. Several studies have noted an association between alcohol consumption and risk of breast cancer [27, 28]. Chen et al. reported that the global distribution of FBC incidence was in line with the consumption of alcohol worldwide [4]. In other word, the FBC incidence was higher in Western Europe, North America, and Australia, in which the average intake of alcohol was higher than that of other regions [29]. Unsurprisingly, in our study, we found that the mortality rate of FBC attributable to alcohol use was higher in regions with high level of alcohol consumption. For example, in Western Europe, the proportion of alcohol related-FBC deaths was as high as 20%. While we observed a decrease of the mortality rate of alcohol related-FBC in these regions, an unfavorable increasing trend was simultaneously seen in most of other regions. More importantly, a recent modeling study reported that the global adult per-capita alcohol consumption increased from 5.9 L to 6.5 L between 1990 and 2017, and is forecasted to reach 7.6 L by 2030 [30]. This prediction indicates that alcohol might be still the major culprit for FBC in the near future and highlights the priority of more practical alcohol control policies. Of note is that the contribution of alcohol in some Asian countries should be interpreted with caution, because alcohol drinking is forbidden in the culture in these Muslim countries.

A similar temporal trend was seen for mortality rate of FBC attributable to tobacco. In our study, we found that the fraction of FBC-related deaths was nearly 5.2%, which was lower than the estimates in previous studies [31]. Tobacco use and exposure to tobacco smoke is one of the major causes of premature mortality and disease worldwide. Fortunately, tobacco use is highly avoidable. Owing to the continuous efforts to combating tobacco, such as the Framework Convention on Tobacco Control, the age-standardized prevalence of daily smoking was seen an remarkable decline for both men and women in a number of countries [32]. However, the battle with tobacco also has a long way to go. Given the persistent increase in the number of smokers worldwide and the recent advent of novel tobacco products, additional potent efforts are warranted to achieve a smoke-free world, help smokers to quit, and particularly protect youth from tobacco use and second-hand tobacco exposure [32].

Of note is the increase of mortality rate of FBC attributable to overweight and diabetes, which indicates an alarming increase of the FBC incidence rate during the past decades. This finding is largely consistent with that of previous studies [33, 34]. For example, Rezende et al. reported that cancer cases attributable to overweight will reach 29,490, which will be 4.6% of all cancers in Brazil in 2025, and the cancer sites contributing most to the number of attributable cases were breast [33]. Saito et al. predicted an increase of 53.3% in incident cancer cases associated with type 2 diabetes in Japanese women between 2010 and 2030. The age-adjusted incidence of FBC will increase from 108.5/100,000 to 145.8/100,000 during the same period [34]. Worldwide, although overweight and diabetes accounts for only < 10% of total FBC deaths, respectively, these proportions have experienced a significant increase during the last three decades. Moreover, this increasing trend was ubiquitous across the world. In recent years, overweight, including obesity, has become a pandemic and imposes an increasing disease burden on human-being [35, 36]. The global increase of overweight prevalence might not only counteract the achievements of current prevention strategies in developed countries, but also exacerbate the disease burden of FBC in developing countries. The keys of strategies to prevent and control obesity lie on many aspects [37]. First, the governments and policy makers should play a leading role in emphasizing the necessity of solving the obesity problem and evoking the public awareness to combat it. Second, the prevention strategies should be multifaceted and involves government, industry, public sector, community, and every people. Third, our government and public sector should allocate more funds and investments specifically to create an environment, in which the healthy lifestyle are largely encouraged [38]. Likewise, the prevalence of diabetes in adults has remarkably increased, or at best remained unchanged, in every country, during the last decades [39]. The prevention schedules designed for obesity are might be proper for diabetes, because obesity and diabetes share most of risk factors.

The limitations of this type of population-based study should be noted here. First, all these five risk factors included in our study can only explain 30.3% of total FBC related deaths. This relatively low proportion might be owing to the dearth of the raw data, because not all FBC cases were reported the major risk factors that they have encountered. Moreover, the contributions of psychological and social risk factors might be concealed and are difficult to quantify. However, since the increasing prevalence of these risk factors, they deserve more attention in the current and future scheme of FBC prevention. Second, the interactions among the environmental risk factors of interest herein and their interactions with genetic variants have not been considered in the GBD study, although these types of interaction were not rarely seen in a single individual [40, 41]. Third, the increasing trend of FBC mortality in certain regions, especially in developed countries, might be ascribed to the increase in FBC screening, resulting in an increase in the FBC diagnosis, report, and record. As a result, the increase in FBC mortality should be interpreted with cautions. Finally, in countries where currently lack of cancer registry, the estimates of FBC disease burden might be biased. However, despite data scarcity and different estimation methods, most GLOBOCAN estimates fell within the 95% UIs of the GBD estimates [42]. This consistency indicates the robustness of the GBD estimates [43]. Although these limitations, our study provides a comprehensive description of FBC-related deaths attributable to the five well-known risk factors at the secular, spatial, and population levels. Our findings are important complements to the previous studies [18, 44].

In conclusion, we report a global decrease and geographically diverse trend of FBC mortality rate. This decrease was mainly driven by the effective control for alcohol and tobacco use. However, we also note a ubiquitous increase in mortality of FBC attributable to overweight and diabetes. Given the global increasing trend in prevalence of obesity and diabetes, more potent and tailored prevention strategies for these two commonly diagnosed complaints, which are critical for FBC prevention, are urgently warranted.

## Supplementary Information

**Additional file 1: Box**. Methods for calculating the FBC mortality from each risk factor. **Table S1**. The estimated average percentage change in female breast cancer mortality by region and risk factors

## Data Availability

The datasets generated and/or analyzed during the current study are available in the GBD study 2017 online repository, [http://ghdx.healthdata.org/gbd-2017].

## Abbreviations

FBC: Female breast cancer
BMI: Body mass index
FPG: High fasting plasma glucose
ASMR: Age-standardized mortality rate
EAPC: Estimated average percentage change
