# Supplementary material for: Global, regional, and national mortality trends of female breast cancer by risk factor, 1990–2017
Source: BMC Cancer. 2021 Apr 24;21:459. doi: 10.1186/s12885-021-08217-5 (PMC8070325; doi:10.1186/s12885-021-08217-5)
Supplement: Supplementary file 1 — Additional file 1: Box. Methods for calculating the FBC mortality from each risk factor. Table S1. The estimated average percentage change in female breast cancer mortality by region and risk factors [file 12885_2021_8217_MOESM1_ESM.docx]

**Supplement material**

**Global, regional, and national mortality trends of female breast cancer by risk factor, 1990-2017**

Authors:

Hui Liu, Wenjie Shi, Zhi Jin, Rui Zhuo, Jie Dong, Qiufeng Lao, Shengle Li, Weiyi Pang

***Box. Methods for calculating the FBC mortality from each risk factor.***

| For high BMI, a systematic review has been conducted to identify studies providing nationally or subnationally representative estimates of overweight prevalence, obesity prevalence or mean BMI. The representative studies providing data on mean BMI or prevalence of overweight or obesity among adults or children were included. For adults, studies were included if they defined overweight as BMI ≥25 kg/m^2^ and obesity as BMI ≥30 kg/m^2^, or if estimates using those cutoffs could be back-calculated from reported categories. For alcohol use, a systematic review of the literature was performed to extract data on primary indicators. The information from randomized controlled trials, cohort, pooled cohort, and case-control studies were firstly collated and then the collected data were used to determine the relative risk for the risk-outcome pairs included in GBD 2017. Physical activity was measured among adults greater than or equal to 25 years of age, for durations of at least ten minutes at a time, across all domains of life (leisure/recreation, work/household and transport). Frequency, duration and intensity of activity was used to calculate total metabolic equivalent minutes per week. MET (Metabolic Equivalent) is the ratio of the working metabolic rate to the resting metabolic rate. People who has <4000 MET-min/week were defined as low physical activity. For tobacco use, primary data were extracted from individual-level microdata and survey report tabulations. The GBD collaborators extracted data on current, former, and/or ever smoked tobacco use reported as any combination of frequency of use (daily, occasional, and unspecified, which includes both daily and occasional smokers) and type of smoked tobacco used (all smoked tobacco, cigarettes, hookah, and other smoked tobacco products such as cigars or pipes), resulting in 36 possible combinations. High FPG is measured as the mean FPG in a population, where FPG is a continuous exposure in units of mmol/L. FPG >126 mg/dL (7 mmol/L) was defined as high FPG. FPG data come from 3 sources: 1) estimates of mean FPG in a representative population; 2) individual-level data of fasting plasma glucose measured from surveys; and 3) estimates of diabetes prevalence in a representative population. More details see appendix of the previous studies [1, 2]. |
| --- |

References

1. **Global, regional, and national comparative risk assessment of 84 behavioural, environmental and occupational, and metabolic risks or clusters of risks for 195 countries and territories, 1990-2017: a systematic analysis for the Global Burden of Disease Study 2017**. *Lancet (London, England)* 2018, **392**(10159):1923-1994.

2. **Global, regional, and national incidence, prevalence, and years lived with disability for 354 diseases and injuries for 195 countries and territories, 1990-2017: a systematic analysis for the Global Burden of Disease Study 2017**. *Lancet* 2018, **392**(10159):1789-1858.

| **Table S1. The estimated average percentage change in female breast cancer mortality**  **by region and risk factors** | | | | | |
| --- | --- | --- | --- | --- | --- |
| **Location** | **Risk factors** | **EAPCS** | **95% CI** |  | **P value** |
| Romania | All cause | 0.14 | -0.02 | 0.3 | 0.074 |
| Romania | Tobacco | -0.14 | -0.3 | 0.03 | 0.11 |
| Romania | Alcohol use | -0.44 | -0.54 | -0.33 | 0 |
| Romania | High fasting plasma glucose | 1.29 | 1.14 | 1.44 | 0 |
| Romania | High body-mass index | 1.96 | 1.68 | 2.24 | 0 |
| Romania | Low physical activity | 0.44 | 0.28 | 0.6 | 0 |
| Global | All cause | -0.59 | -0.66 | -0.52 | 0 |
| Global | Tobacco | -1.77 | -1.85 | -1.7 | 0 |
| Global | Alcohol use | -1.73 | -1.82 | -1.63 | 0 |
| Global | High fasting plasma glucose | 0.26 | 0.23 | 0.3 | 0 |
| Global | High body-mass index | 1.26 | 1.22 | 1.3 | 0 |
| Global | Low physical activity | -0.69 | -0.76 | -0.63 | 0 |
| Oman | All cause | 0.69 | 0.56 | 0.82 | 0 |
| Oman | Tobacco | 0.35 | 0.24 | 0.46 | 0 |
| Oman | Alcohol use | 1.85 | 1.26 | 2.44 | 0 |
| Oman | High fasting plasma glucose | 2 | 1.87 | 2.12 | 0 |
| Oman | High body-mass index | 6.42 | 5.97 | 6.88 | 0 |
| Oman | Low physical activity | 0.78 | 0.65 | 0.91 | 0 |
| Solomon Islands | All cause | 0.8 | 0.76 | 0.84 | 0 |
| Solomon Islands | Tobacco | 0.8 | 0.75 | 0.85 | 0 |
| Solomon Islands | Alcohol use | 3.26 | 2.83 | 3.69 | 0 |
| Solomon Islands | High fasting plasma glucose | 1.87 | 1.8 | 1.95 | 0 |
| Solomon Islands | High body-mass index | 1.59 | 1.16 | 2.02 | 0 |
| Solomon Islands | Low physical activity | 0.83 | 0.79 | 0.87 | 0 |
| Costa Rica | All cause | 0.3 | 0.13 | 0.48 | 0.001 |
| Costa Rica | Tobacco | -0.81 | -0.99 | -0.63 | 0 |
| Costa Rica | Alcohol use | -0.29 | -0.49 | -0.09 | 0.006 |
| Costa Rica | High fasting plasma glucose | 0.39 | 0.22 | 0.56 | 0 |
| Costa Rica | High body-mass index | 2.27 | 2.04 | 2.49 | 0 |
| Costa Rica | Low physical activity | 0.4 | 0.23 | 0.57 | 0 |
| Greenland | All cause | -2.21 | -2.41 | -2.02 | 0 |
| Greenland | Tobacco | -2.41 | -2.58 | -2.23 | 0 |
| Greenland | Alcohol use | -2.12 | -2.3 | -1.95 | 0 |
| Greenland | High fasting plasma glucose | -3.11 | -3.31 | -2.9 | 0 |
| Greenland | High body-mass index | -1.18 | -1.43 | -0.93 | 0 |
| Greenland | Low physical activity | -2.13 | -2.32 | -1.94 | 0 |
| North Korea | All cause | 0.99 | 0.91 | 1.06 | 0 |
| North Korea | Tobacco | 0.53 | 0.42 | 0.64 | 0 |
| North Korea | Alcohol use | 0.12 | -0.09 | 0.32 | 0.252 |
| North Korea | High fasting plasma glucose | 1.88 | 1.74 | 2.02 | 0 |
| North Korea | High body-mass index | -0.03 | -0.18 | 0.12 | 0.683 |
| North Korea | Low physical activity | 1.06 | 0.98 | 1.15 | 0 |
| Serbia | All cause | 0.75 | 0.63 | 0.87 | 0 |
| Serbia | Tobacco | 1.01 | 0.84 | 1.17 | 0 |
| Serbia | Alcohol use | 0.94 | 0.75 | 1.14 | 0 |
| Serbia | High fasting plasma glucose | 2.17 | 2.02 | 2.33 | 0 |
| Serbia | High body-mass index | 2.72 | 2.45 | 2.98 | 0 |
| Serbia | Low physical activity | 1.09 | 0.97 | 1.2 | 0 |
| Saudi Arabia | All cause | 1.49 | 1.3 | 1.69 | 0 |
| Saudi Arabia | Tobacco | 2.28 | 1.98 | 2.57 | 0 |
| Saudi Arabia | Alcohol use | 0.5 | 0.25 | 0.75 | 0 |
| Saudi Arabia | High fasting plasma glucose | 2.53 | 2.15 | 2.9 | 0 |
| Saudi Arabia | High body-mass index | 4.77 | 4.15 | 5.4 | 0 |
| Saudi Arabia | Low physical activity | 1.63 | 1.43 | 1.84 | 0 |
| China | All cause | -0.08 | -0.22 | 0.07 | 0.293 |

| China | Tobacco | -0.52 | -0.64 | -0.39 | 0 |
| --- | --- | --- | --- | --- | --- |
| China | Alcohol use | 0.48 | 0.18 | 0.78 | 0.003 |
| China | High fasting plasma glucose | 0.93 | 0.67 | 1.18 | 0 |
| China | High body-mass index | 3.71 | 3.54 | 3.88 | 0 |
| China | Low physical activity | 0.18 | 0.04 | 0.33 | 0.017 |
| Vanuatu | All cause | 0.92 | 0.87 | 0.97 | 0 |
| Vanuatu | Tobacco | 0.39 | 0.32 | 0.46 | 0 |
| Vanuatu | Alcohol use | -1.57 | -1.68 | -1.46 | 0 |
| Vanuatu | High fasting plasma glucose | 2.24 | 2.15 | 2.33 | 0 |
| Vanuatu | High body-mass index | 1.33 | 1.21 | 1.45 | 0 |
| Vanuatu | Low physical activity | 1.01 | 0.96 | 1.06 | 0 |
| El Salvador | All cause | 1.24 | 1.1 | 1.39 | 0 |
| El Salvador | Tobacco | 0.86 | 0.73 | 0.98 | 0 |
| El Salvador | Alcohol use | 2.16 | 1.88 | 2.45 | 0 |
| El Salvador | High fasting plasma glucose | 2.24 | 2.05 | 2.44 | 0 |
| El Salvador | High body-mass index | 2.92 | 2.47 | 3.38 | 0 |
| El Salvador | Low physical activity | 1.35 | 1.2 | 1.51 | 0 |
| Madagascar | All cause | -0.18 | -0.28 | -0.08 | 0.001 |
| Madagascar | Tobacco | -0.78 | -0.87 | -0.69 | 0 |
| Madagascar | Alcohol use | -1.61 | -2.05 | -1.18 | 0 |
| Madagascar | High fasting plasma glucose | 0.6 | 0.5 | 0.69 | 0 |
| Madagascar | High body-mass index | 2.52 | 2.28 | 2.76 | 0 |
| Madagascar | Low physical activity | -0.15 | -0.25 | -0.06 | 0.002 |
| Venezuela | All cause | 0.56 | 0.39 | 0.73 | 0 |
| Venezuela | Tobacco | -0.61 | -0.78 | -0.44 | 0 |
| Venezuela | Alcohol use | -0.12 | -0.34 | 0.09 | 0.254 |
| Venezuela | High fasting plasma glucose | 0.92 | 0.64 | 1.2 | 0 |
| Venezuela | High body-mass index | 2.19 | 2.01 | 2.36 | 0 |
| Venezuela | Low physical activity | 0.64 | 0.47 | 0.82 | 0 |
| Georgia | All cause | 0.51 | -0.1 | 1.12 | 0.095 |
| Georgia | Tobacco | 1.1 | 0.46 | 1.74 | 0.002 |
| Georgia | Alcohol use | 0.8 | -0.09 | 1.71 | 0.077 |
| Georgia | High fasting plasma glucose | 2.19 | 1.51 | 2.89 | 0 |
| Georgia | High body-mass index | 2.64 | 2 | 3.28 | 0 |
| Georgia | Low physical activity | 0.72 | 0.11 | 1.34 | 0.023 |
| High SDI | All cause | -1.46 | -1.51 | -1.4 | 0 |
| High SDI | Tobacco | -2.58 | -2.64 | -2.52 | 0 |
| High SDI | Alcohol use | -1.88 | -1.93 | -1.83 | 0 |
| High SDI | High fasting plasma glucose | -0.37 | -0.49 | -0.25 | 0 |
| High SDI | High body-mass index | -0.19 | -0.25 | -0.13 | 0 |
| High SDI | Low physical activity | -1.43 | -1.48 | -1.37 | 0 |
| Burundi | All cause | -1.06 | -1.23 | -0.89 | 0 |
| Burundi | Tobacco | -1.06 | -1.25 | -0.87 | 0 |
| Burundi | Alcohol use | -1.31 | -1.5 | -1.13 | 0 |
| Burundi | High fasting plasma glucose | -0.51 | -0.66 | -0.35 | 0 |
| Burundi | High body-mass index | 0.24 | 0.1 | 0.38 | 0.002 |
| Burundi | Low physical activity | -0.99 | -1.15 | -0.83 | 0 |
| Cambodia | All cause | 0.4 | 0.28 | 0.51 | 0 |
| Cambodia | Tobacco | -0.12 | -0.26 | 0.01 | 0.063 |
| Cambodia | Alcohol use | 2.92 | 2.19 | 3.65 | 0 |
| Cambodia | High fasting plasma glucose | 0.72 | 0.58 | 0.87 | 0 |
| Cambodia | High body-mass index | 3.06 | 2.96 | 3.17 | 0 |
| Cambodia | Low physical activity | 0.45 | 0.35 | 0.56 | 0 |
| Qatar | All cause | -0.3 | -0.43 | -0.18 | 0 |
| Qatar | Tobacco | -0.16 | -0.34 | 0.02 | 0.077 |
| Qatar | Alcohol use | 0.21 | -0.31 | 0.74 | 0.416 |
| Qatar | High fasting plasma glucose | -0.25 | -0.62 | 0.12 | 0.173 |
| Qatar | High body-mass index | 0.73 | 0.47 | 0.99 | 0 |

| Qatar | Low physical activity | -0.29 | -0.41 | -0.16 | 0 |
| --- | --- | --- | --- | --- | --- |
| Yemen | All cause | 1.18 | 1.16 | 1.21 | 0 |
| Yemen | Tobacco | 1.14 | 1.12 | 1.17 | 0 |
| Yemen | Alcohol use | -4.41 | -5.1 | -3.73 | 0 |
| Yemen | High fasting plasma glucose | 1.98 | 1.88 | 2.08 | 0 |
| Yemen | High body-mass index | 3.73 | 3.57 | 3.88 | 0 |
| Yemen | Low physical activity | 1.22 | 1.2 | 1.23 | 0 |
| Guatemala | All cause | -0.07 | -0.32 | 0.19 | 0.6 |
| Guatemala | Tobacco | -0.44 | -0.68 | -0.2 | 0.001 |
| Guatemala | Alcohol use | -0.25 | -0.51 | 0 | 0.052 |
| Guatemala | High fasting plasma glucose | 1.44 | 1.12 | 1.77 | 0 |
| Guatemala | High body-mass index | 3.22 | 2.82 | 3.62 | 0 |
| Guatemala | Low physical activity | 0.05 | -0.2 | 0.3 | 0.689 |
| Ghana | All cause | 0.51 | 0.44 | 0.58 | 0 |
| Ghana | Tobacco | 0.27 | 0.1 | 0.44 | 0.003 |
| Ghana | Alcohol use | 0.67 | 0.51 | 0.83 | 0 |
| Ghana | High fasting plasma glucose | 1.78 | 1.69 | 1.88 | 0 |
| Ghana | High body-mass index | 3.21 | 2.93 | 3.48 | 0 |
| Ghana | Low physical activity | 0.67 | 0.6 | 0.74 | 0 |
| Slovenia | All cause | -1.32 | -1.62 | -1.02 | 0 |
| Slovenia | Tobacco | -2.32 | -2.59 | -2.05 | 0 |
| Slovenia | Alcohol use | -3.47 | -3.95 | -2.99 | 0 |
| Slovenia | High fasting plasma glucose | -0.82 | -1.25 | -0.39 | 0.001 |
| Slovenia | High body-mass index | -0.01 | -0.5 | 0.5 | 0.982 |
| Slovenia | Low physical activity | -1.04 | -1.34 | -0.75 | 0 |
| Slovakia | All cause | -0.37 | -0.49 | -0.25 | 0 |
| Slovakia | Tobacco | -1.61 | -1.79 | -1.44 | 0 |
| Slovakia | Alcohol use | -0.92 | -1.11 | -0.73 | 0 |
| Slovakia | High fasting plasma glucose | 0.31 | 0.18 | 0.45 | 0 |
| Slovakia | High body-mass index | 0.84 | 0.61 | 1.07 | 0 |
| Slovakia | Low physical activity | -0.17 | -0.29 | -0.05 | 0.008 |
| Syria | All cause | 0.15 | -0.12 | 0.42 | 0.274 |
| Syria | Tobacco | -0.56 | -0.8 | -0.31 | 0 |
| Syria | Alcohol use | 0.49 | 0.3 | 0.68 | 0 |
| Syria | High fasting plasma glucose | 0.87 | 0.51 | 1.23 | 0 |
| Syria | High body-mass index | 2.37 | 2.21 | 2.54 | 0 |
| Syria | Low physical activity | 0.26 | -0.02 | 0.54 | 0.064 |
| Guinea | All cause | 0.97 | 0.86 | 1.09 | 0 |
| Guinea | Tobacco | 0.74 | 0.6 | 0.87 | 0 |
| Guinea | Alcohol use | 0.57 | 0.32 | 0.83 | 0 |
| Guinea | High fasting plasma glucose | 2.58 | 2.35 | 2.81 | 0 |
| Guinea | High body-mass index | 2.51 | 2.28 | 2.73 | 0 |
| Guinea | Low physical activity | 1.03 | 0.89 | 1.17 | 0 |
| Guam | All cause | 0.87 | 0.62 | 1.12 | 0 |
| Guam | Tobacco | 0.57 | 0.34 | 0.8 | 0 |
| Guam | Alcohol use | 2.33 | 2.17 | 2.48 | 0 |
| Guam | High fasting plasma glucose | 1.52 | 1.2 | 1.84 | 0 |
| Guam | High body-mass index | 1.5 | 1.27 | 1.72 | 0 |
| Guam | Low physical activity | 0.87 | 0.62 | 1.13 | 0 |
| United States | All cause | -1.77 | -1.89 | -1.66 | 0 |
| United States | Tobacco | -3.28 | -3.38 | -3.18 | 0 |
| United States | Alcohol use | -1.63 | -1.74 | -1.53 | 0 |
| United States | High fasting plasma glucose | 0.42 | 0.06 | 0.78 | 0.023 |
| United States | High body-mass index | -0.66 | -0.75 | -0.57 | 0 |
| United States | Low physical activity | -1.84 | -1.96 | -1.72 | 0 |
| Tonga | All cause | 0.01 | -0.08 | 0.1 | 0.757 |
| Tonga | Tobacco | -0.23 | -0.33 | -0.13 | 0 |
| Tonga | Alcohol use | 1.93 | 1.47 | 2.4 | 0 |

| Tonga | High fasting plasma glucose | 0.65 | 0.57 | 0.73 | 0 |
| --- | --- | --- | --- | --- | --- |
| Tonga | High body-mass index | 0.41 | 0.03 | 0.78 | 0.034 |
| Tonga | Low physical activity | 0.04 | -0.04 | 0.13 | 0.322 |
| Indonesia | All cause | -0.49 | -0.62 | -0.36 | 0 |
| Indonesia | Tobacco | -0.15 | -0.31 | 0.02 | 0.08 |
| Indonesia | Alcohol use | 0.66 | 0.55 | 0.78 | 0 |
| Indonesia | High fasting plasma glucose | 0.74 | 0.56 | 0.92 | 0 |
| Indonesia | High body-mass index | 2.69 | 2.58 | 2.79 | 0 |
| Indonesia | Low physical activity | -0.42 | -0.55 | -0.29 | 0 |
| Cameroon | All cause | 0.27 | 0.17 | 0.37 | 0 |
| Cameroon | Tobacco | -0.3 | -0.42 | -0.18 | 0 |
| Cameroon | Alcohol use | 0.48 | 0.45 | 0.52 | 0 |
| Cameroon | High fasting plasma glucose | 1.74 | 1.61 | 1.87 | 0 |
| Cameroon | High body-mass index | 0.8 | 0.64 | 0.96 | 0 |
| Cameroon | Low physical activity | 0.43 | 0.34 | 0.53 | 0 |
| Laos | All cause | -0.15 | -0.24 | -0.06 | 0.003 |
| Laos | Tobacco | -0.38 | -0.51 | -0.25 | 0 |
| Laos | Alcohol use | -0.24 | -0.34 | -0.13 | 0 |
| Laos | High fasting plasma glucose | 0.73 | 0.58 | 0.89 | 0 |
| Laos | High body-mass index | 3.58 | 3.43 | 3.73 | 0 |
| Laos | Low physical activity | -0.11 | -0.2 | -0.01 | 0.028 |
| Mauritius | All cause | 2.62 | 2.36 | 2.87 | 0 |
| Mauritius | Tobacco | 2.18 | 1.97 | 2.39 | 0 |
| Mauritius | Alcohol use | 2.38 | 2.11 | 2.65 | 0 |
| Mauritius | High fasting plasma glucose | 4.8 | 4.39 | 5.22 | 0 |
| Mauritius | High body-mass index | 3.8 | 3.54 | 4.06 | 0 |
| Mauritius | Low physical activity | 2.67 | 2.42 | 2.93 | 0 |
| Kazakhstan | All cause | 0.29 | -0.26 | 0.85 | 0.289 |
| Kazakhstan | Tobacco | 0.25 | -0.19 | 0.69 | 0.25 |
| Kazakhstan | Alcohol use | 0.44 | -0.13 | 1.02 | 0.128 |
| Kazakhstan | High fasting plasma glucose | 0.86 | 0.26 | 1.45 | 0.006 |
| Kazakhstan | High body-mass index | 2.65 | 2.01 | 3.3 | 0 |
| Kazakhstan | Low physical activity | 0.57 | 0.02 | 1.13 | 0.043 |
| Taiwan (Province of China) | All cause | 1.25 | 1.09 | 1.42 | 0 |
| Taiwan (Province of China) | Tobacco | 0.13 | -0.15 | 0.41 | 0.344 |
| Taiwan (Province of China) | Alcohol use | 2.3 | 2.1 | 2.51 | 0 |
| Taiwan (Province of China) | High fasting plasma glucose | 1.9 | 1.55 | 2.25 | 0 |
| Taiwan (Province of China) | High body-mass index | 2.84 | 2.5 | 3.18 | 0 |
| Taiwan (Province of China) | Low physical activity | 1.48 | 1.31 | 1.65 | 0 |
| Mexico | All cause | 0.2 | 0.1 | 0.29 | 0 |
| Mexico | Tobacco | -1.49 | -1.65 | -1.33 | 0 |
| Mexico | Alcohol use | -0.71 | -0.96 | -0.46 | 0 |
| Mexico | High fasting plasma glucose | 0.41 | 0.24 | 0.58 | 0 |
| Mexico | High body-mass index | 1.82 | 1.58 | 2.05 | 0 |
| Mexico | Low physical activity | 0.16 | 0.06 | 0.25 | 0.002 |
| Malawi | All cause | -0.66 | -1 | -0.32 | 0.001 |
| Malawi | Tobacco | -1.05 | -1.42 | -0.68 | 0 |
| Malawi | Alcohol use | -0.18 | -0.52 | 0.17 | 0.302 |
| Malawi | High fasting plasma glucose | -0.23 | -0.57 | 0.12 | 0.19 |
| Malawi | High body-mass index | 6.19 | 5.61 | 6.77 | 0 |
| Malawi | Low physical activity | -0.57 | -0.88 | -0.25 | 0.001 |
| Dominica | All cause | 0.73 | 0.63 | 0.84 | 0 |
| Dominica | Tobacco | 0.27 | 0.2 | 0.35 | 0 |
| Dominica | Alcohol use | 0.27 | 0.04 | 0.5 | 0.021 |
| Dominica | High fasting plasma glucose | 0.91 | 0.84 | 0.98 | 0 |
| Dominica | High body-mass index | 1.95 | 1.83 | 2.06 | 0 |
| Dominica | Low physical activity | 0.78 | 0.69 | 0.87 | 0 |
| Belize | All cause | 0.83 | 0.54 | 1.12 | 0 |

| Belize | Tobacco | 0.36 | 0.09 | 0.64 | 0.012 |
| --- | --- | --- | --- | --- | --- |
| Belize | Alcohol use | 2.26 | 2.01 | 2.52 | 0 |
| Belize | High fasting plasma glucose | 1.52 | 1.05 | 1.99 | 0 |
| Belize | High body-mass index | 1.97 | 1.34 | 2.61 | 0 |
| Belize | Low physical activity | 0.81 | 0.52 | 1.1 | 0 |
| Tunisia | All cause | 0.01 | -0.2 | 0.23 | 0.915 |
| Tunisia | Tobacco | -0.63 | -0.91 | -0.36 | 0 |
| Tunisia | Alcohol use | 0.24 | -0.02 | 0.5 | 0.069 |
| Tunisia | High fasting plasma glucose | 0.68 | 0.39 | 0.97 | 0 |
| Tunisia | High body-mass index | 0.77 | 0.44 | 1.11 | 0 |
| Tunisia | Low physical activity | 0.03 | -0.18 | 0.24 | 0.746 |
| Northern Mariana Islands | All cause | -0.4 | -0.62 | -0.18 | 0.001 |
| Northern Mariana Islands | Tobacco | -0.82 | -1.05 | -0.58 | 0 |
| Northern Mariana Islands | Alcohol use | 0.38 | 0.18 | 0.59 | 0.001 |
| Northern Mariana Islands | High fasting plasma glucose | 0.22 | 0.05 | 0.4 | 0.012 |
| Northern Mariana Islands | High body-mass index | 0.03 | -0.11 | 0.18 | 0.641 |
| Northern Mariana Islands | Low physical activity | -0.38 | -0.59 | -0.16 | 0.001 |
| Kyrgyzstan | All cause | -1.35 | -1.53 | -1.18 | 0 |
| Kyrgyzstan | Tobacco | -1.08 | -1.32 | -0.83 | 0 |
| Kyrgyzstan | Alcohol use | -1.66 | -1.83 | -1.48 | 0 |
| Kyrgyzstan | High fasting plasma glucose | -1.08 | -1.22 | -0.94 | 0 |
| Kyrgyzstan | High body-mass index | -0.28 | -0.59 | 0.04 | 0.083 |
| Kyrgyzstan | Low physical activity | -1.16 | -1.34 | -0.98 | 0 |
| Chad | All cause | 0.8 | 0.75 | 0.84 | 0 |
| Chad | Tobacco | 0.91 | 0.86 | 0.95 | 0 |
| Chad | Alcohol use | 1.23 | 1.2 | 1.26 | 0 |
| Chad | High fasting plasma glucose | 2.19 | 2.13 | 2.26 | 0 |
| Chad | High body-mass index | 2.76 | 2.43 | 3.09 | 0 |
| Chad | Low physical activity | 0.86 | 0.82 | 0.91 | 0 |
| Mozambique | All cause | 0.26 | -0.14 | 0.67 | 0.195 |
| Mozambique | Tobacco | 0.85 | 0.5 | 1.2 | 0 |
| Mozambique | Alcohol use | 2.83 | 2.69 | 2.97 | 0 |
| Mozambique | High fasting plasma glucose | 1.27 | 0.85 | 1.69 | 0 |
| Mozambique | High body-mass index | 6.69 | 4.99 | 8.42 | 0 |
| Mozambique | Low physical activity | 0.33 | -0.06 | 0.72 | 0.097 |
| Guinea-Bissau | All cause | 0.86 | 0.8 | 0.92 | 0 |
| Guinea-Bissau | Tobacco | 0.55 | 0.47 | 0.62 | 0 |
| Guinea-Bissau | Alcohol use | 0.57 | 0.46 | 0.68 | 0 |
| Guinea-Bissau | High fasting plasma glucose | 2.38 | 2.29 | 2.47 | 0 |
| Guinea-Bissau | High body-mass index | 2.32 | 2.1 | 2.54 | 0 |
| Guinea-Bissau | Low physical activity | 1.01 | 0.95 | 1.06 | 0 |
| Dominican Republic | All cause | 2.2 | 2.04 | 2.37 | 0 |
| Dominican Republic | Tobacco | 1.61 | 1.41 | 1.8 | 0 |
| Dominican Republic | Alcohol use | 2.56 | 2.35 | 2.77 | 0 |
| Dominican Republic | High fasting plasma glucose | 3.13 | 2.92 | 3.35 | 0 |
| Dominican Republic | High body-mass index | 3.56 | 3.36 | 3.76 | 0 |
| Dominican Republic | Low physical activity | 2.19 | 2.03 | 2.35 | 0 |
| Turkey | All cause | -0.81 | -1.28 | -0.34 | 0.002 |
| Turkey | Tobacco | -1.66 | -2.12 | -1.2 | 0 |
| Turkey | Alcohol use | -1.47 | -2.2 | -0.73 | 0 |
| Turkey | High fasting plasma glucose | -0.58 | -0.96 | -0.19 | 0.005 |
| Turkey | High body-mass index | -0.23 | -0.8 | 0.35 | 0.428 |
| Turkey | Low physical activity | -0.7 | -1.17 | -0.23 | 0.005 |
| Belarus | All cause | -1.07 | -1.49 | -0.64 | 0 |
| Belarus | Tobacco | -1.51 | -1.91 | -1.11 | 0 |
| Belarus | Alcohol use | -0.7 | -1.11 | -0.27 | 0.002 |
| Belarus | High fasting plasma glucose | -0.77 | -1.23 | -0.32 | 0.002 |
| Belarus | High body-mass index | 1.6 | 0.98 | 2.22 | 0 |

| Belarus | Low physical activity | -0.79 | -1.22 | -0.36 | 0.001 |
| --- | --- | --- | --- | --- | --- |
| Estonia | All cause | -1.14 | -1.45 | -0.83 | 0 |
| Estonia | Tobacco | -2.07 | -2.41 | -1.74 | 0 |
| Estonia | Alcohol use | 1.24 | 0.55 | 1.94 | 0.001 |
| Estonia | High fasting plasma glucose | 0.13 | -0.22 | 0.48 | 0.459 |
| Estonia | High body-mass index | 1.3 | 0.93 | 1.67 | 0 |
| Estonia | Low physical activity | -0.74 | -1.01 | -0.46 | 0 |
| Eritrea | All cause | 0.64 | 0.58 | 0.69 | 0 |
| Eritrea | Tobacco | 0.15 | 0.01 | 0.29 | 0.039 |
| Eritrea | Alcohol use | 0.25 | -0.42 | 0.92 | 0.455 |
| Eritrea | High fasting plasma glucose | 1.51 | 1.47 | 1.55 | 0 |
| Eritrea | High body-mass index | 4.91 | 4.48 | 5.35 | 0 |
| Eritrea | Low physical activity | 0.69 | 0.63 | 0.75 | 0 |
| Afghanistan | All cause | 0.48 | 0.41 | 0.55 | 0 |
| Afghanistan | Tobacco | 1.05 | 0.97 | 1.13 | 0 |
| Afghanistan | Alcohol use | 2.05 | -0.33 | 4.49 | 0.089 |
| Afghanistan | High fasting plasma glucose | 1.34 | 1.23 | 1.45 | 0 |
| Afghanistan | High body-mass index | 4.46 | 3.7 | 5.23 | 0 |
| Afghanistan | Low physical activity | 0.48 | 0.43 | 0.54 | 0 |
| Barbados | All cause | 0.38 | 0.28 | 0.48 | 0 |
| Barbados | Tobacco | -0.41 | -0.53 | -0.29 | 0 |
| Barbados | Alcohol use | 0.37 | 0.22 | 0.52 | 0 |
| Barbados | High fasting plasma glucose | 0.66 | 0.54 | 0.78 | 0 |
| Barbados | High body-mass index | 1.11 | 0.91 | 1.32 | 0 |
| Barbados | Low physical activity | 0.42 | 0.32 | 0.52 | 0 |
| Bhutan | All cause | -0.63 | -0.74 | -0.52 | 0 |
| Bhutan | Tobacco | -1.1 | -1.2 | -0.99 | 0 |
| Bhutan | Alcohol use | -1.69 | -1.94 | -1.43 | 0 |
| Bhutan | High fasting plasma glucose | 0.03 | -0.08 | 0.14 | 0.579 |
| Bhutan | High body-mass index | 4.06 | 3.53 | 4.59 | 0 |
| Bhutan | Low physical activity | -0.44 | -0.54 | -0.34 | 0 |
| Malaysia | All cause | 0.39 | 0.27 | 0.51 | 0 |
| Malaysia | Tobacco | 0.24 | 0.11 | 0.37 | 0.001 |
| Malaysia | Alcohol use | -0.01 | -0.43 | 0.41 | 0.953 |
| Malaysia | High fasting plasma glucose | 0.91 | 0.76 | 1.06 | 0 |
| Malaysia | High body-mass index | 2.54 | 2.33 | 2.75 | 0 |
| Malaysia | Low physical activity | 0.44 | 0.32 | 0.55 | 0 |
| The Gambia | All cause | 1.51 | 1.47 | 1.56 | 0 |
| The Gambia | Tobacco | 1.34 | 1.25 | 1.43 | 0 |
| The Gambia | Alcohol use | 1.31 | 0.81 | 1.81 | 0 |
| The Gambia | High fasting plasma glucose | 2.96 | 2.85 | 3.06 | 0 |
| The Gambia | High body-mass index | 2.91 | 2.65 | 3.17 | 0 |
| The Gambia | Low physical activity | 1.58 | 1.53 | 1.63 | 0 |
| Armenia | All cause | -0.05 | -0.36 | 0.27 | 0.769 |
| Armenia | Tobacco | -0.43 | -0.71 | -0.15 | 0.004 |
| Armenia | Alcohol use | -0.51 | -0.86 | -0.17 | 0.005 |
| Armenia | High fasting plasma glucose | 1.12 | 0.63 | 1.62 | 0 |
| Armenia | High body-mass index | 3.29 | 2.79 | 3.8 | 0 |
| Armenia | Low physical activity | 0.35 | 0.05 | 0.64 | 0.023 |
| Ethiopia | All cause | -0.75 | -0.92 | -0.58 | 0 |
| Ethiopia | Tobacco | -1.02 | -1.24 | -0.8 | 0 |
| Ethiopia | Alcohol use | 0.11 | 0.04 | 0.19 | 0.006 |
| Ethiopia | High fasting plasma glucose | -0.8 | -0.89 | -0.72 | 0 |
| Ethiopia | High body-mass index | 3.93 | 3.59 | 4.27 | 0 |
| Ethiopia | Low physical activity | -0.61 | -0.76 | -0.46 | 0 |
| Brazil | All cause | -0.37 | -0.57 | -0.17 | 0.001 |
| Brazil | Tobacco | -1.69 | -2.06 | -1.32 | 0 |
| Brazil | Alcohol use | 0.06 | -0.37 | 0.49 | 0.788 |

| Brazil | High fasting plasma glucose | -0.8 | -1.08 | -0.52 | 0 |
| --- | --- | --- | --- | --- | --- |
| Brazil | High body-mass index | 1.29 | 0.93 | 1.65 | 0 |
| Brazil | Low physical activity | -0.36 | -0.56 | -0.17 | 0.001 |
| Cape Verde | All cause | 1.09 | 0.97 | 1.22 | 0 |
| Cape Verde | Tobacco | 1.01 | 0.88 | 1.14 | 0 |
| Cape Verde | Alcohol use | 1.26 | 1.18 | 1.35 | 0 |
| Cape Verde | High fasting plasma glucose | 3.15 | 3.07 | 3.23 | 0 |
| Cape Verde | High body-mass index | 2.8 | 2.62 | 2.99 | 0 |
| Cape Verde | Low physical activity | 1.33 | 1.22 | 1.45 | 0 |
| Mongolia | All cause | -0.29 | -0.74 | 0.16 | 0.194 |
| Mongolia | Tobacco | -0.69 | -1.19 | -0.19 | 0.009 |
| Mongolia | Alcohol use | 1.76 | 1.28 | 2.25 | 0 |
| Mongolia | High fasting plasma glucose | 0.3 | -0.14 | 0.74 | 0.175 |
| Mongolia | High body-mass index | 1.07 | 0.75 | 1.39 | 0 |
| Mongolia | Low physical activity | -0.05 | -0.48 | 0.37 | 0.796 |
| Puerto Rico | All cause | -0.51 | -0.65 | -0.37 | 0 |
| Puerto Rico | Tobacco | -1.06 | -1.2 | -0.91 | 0 |
| Puerto Rico | Alcohol use | -0.64 | -0.86 | -0.42 | 0 |
| Puerto Rico | High fasting plasma glucose | 0.26 | 0.07 | 0.44 | 0.009 |
| Puerto Rico | High body-mass index | 1.15 | 0.86 | 1.43 | 0 |
| Puerto Rico | Low physical activity | -0.54 | -0.68 | -0.39 | 0 |
| Cuba | All cause | -0.12 | -0.27 | 0.02 | 0.099 |
| Cuba | Tobacco | -0.67 | -0.78 | -0.56 | 0 |
| Cuba | Alcohol use | 0.5 | 0.31 | 0.69 | 0 |
| Cuba | High fasting plasma glucose | -0.11 | -0.37 | 0.15 | 0.401 |
| Cuba | High body-mass index | 1.88 | 1.51 | 2.25 | 0 |
| Cuba | Low physical activity | -0.05 | -0.21 | 0.1 | 0.506 |
| Myanmar | All cause | -0.78 | -0.98 | -0.58 | 0 |
| Myanmar | Tobacco | -1.08 | -1.29 | -0.87 | 0 |
| Myanmar | Alcohol use | 1.46 | 1.16 | 1.77 | 0 |
| Myanmar | High fasting plasma glucose | -0.37 | -0.51 | -0.22 | 0 |
| Myanmar | High body-mass index | 3.69 | 3.38 | 3.99 | 0 |
| Myanmar | Low physical activity | -0.63 | -0.82 | -0.43 | 0 |
| Cote d'Ivoire | All cause | 0.63 | 0.54 | 0.72 | 0 |
| Cote d'Ivoire | Tobacco | 0.98 | 0.89 | 1.07 | 0 |
| Cote d'Ivoire | Alcohol use | 0.03 | -0.27 | 0.32 | 0.858 |
| Cote d'Ivoire | High fasting plasma glucose | 2.08 | 1.88 | 2.28 | 0 |
| Cote d'Ivoire | High body-mass index | 1.62 | 1.51 | 1.73 | 0 |
| Cote d'Ivoire | Low physical activity | 0.7 | 0.61 | 0.78 | 0 |
| Paraguay | All cause | 1.17 | 0.96 | 1.38 | 0 |
| Paraguay | Tobacco | 1 | 0.66 | 1.34 | 0 |
| Paraguay | Alcohol use | -0.07 | -0.31 | 0.16 | 0.513 |
| Paraguay | High fasting plasma glucose | 2.37 | 2.04 | 2.7 | 0 |
| Paraguay | High body-mass index | 3.3 | 3 | 3.6 | 0 |
| Paraguay | Low physical activity | 1.28 | 1.07 | 1.49 | 0 |
| Latvia | All cause | -0.2 | -0.53 | 0.14 | 0.237 |
| Latvia | Tobacco | -0.17 | -0.47 | 0.13 | 0.246 |
| Latvia | Alcohol use | 0.76 | 0.35 | 1.18 | 0.001 |
| Latvia | High fasting plasma glucose | 1.24 | 0.91 | 1.56 | 0 |
| Latvia | High body-mass index | 2.35 | 1.99 | 2.71 | 0 |
| Latvia | Low physical activity | 0.12 | -0.19 | 0.43 | 0.434 |
| Liberia | All cause | 0.81 | 0.65 | 0.97 | 0 |
| Liberia | Tobacco | 0.31 | 0.14 | 0.48 | 0.001 |
| Liberia | Alcohol use | 0.38 | 0.14 | 0.62 | 0.003 |
| Liberia | High fasting plasma glucose | 2.25 | 2.13 | 2.37 | 0 |
| Liberia | High body-mass index | 2.98 | 2.44 | 3.52 | 0 |
| Liberia | Low physical activity | 0.9 | 0.73 | 1.07 | 0 |
| Maldives | All cause | -1.92 | -2.25 | -1.59 | 0 |

| Maldives | Tobacco | -1.42 | -1.72 | -1.11 | 0 |
| --- | --- | --- | --- | --- | --- |
| Maldives | Alcohol use | -0.8 | -1.81 | 0.23 | 0.121 |
| Maldives | High fasting plasma glucose | -1.72 | -2.07 | -1.37 | 0 |
| Maldives | High body-mass index | 2.93 | 2.73 | 3.13 | 0 |
| Maldives | Low physical activity | -1.7 | -2.04 | -1.36 | 0 |
| South Sudan | All cause | -0.56 | -0.74 | -0.37 | 0 |
| South Sudan | Tobacco | -0.53 | -0.7 | -0.35 | 0 |
| South Sudan | Alcohol use | -0.31 | -0.52 | -0.1 | 0.006 |
| South Sudan | High fasting plasma glucose | 0.13 | -0.04 | 0.3 | 0.126 |
| South Sudan | High body-mass index | 1.23 | 0.99 | 1.47 | 0 |
| South Sudan | Low physical activity | -0.55 | -0.72 | -0.38 | 0 |
| Rwanda | All cause | -0.92 | -1.09 | -0.76 | 0 |
| Rwanda | Tobacco | -0.58 | -0.74 | -0.42 | 0 |
| Rwanda | Alcohol use | -1.59 | -1.79 | -1.39 | 0 |
| Rwanda | High fasting plasma glucose | -0.71 | -0.89 | -0.52 | 0 |
| Rwanda | High body-mass index | 3.78 | 3.46 | 4.1 | 0 |
| Rwanda | Low physical activity | -0.81 | -0.95 | -0.66 | 0 |
| India | All cause | 0.85 | 0.58 | 1.12 | 0 |
| India | Tobacco | -0.21 | -0.52 | 0.1 | 0.177 |
| India | Alcohol use | 1.63 | 1.4 | 1.86 | 0 |
| India | High fasting plasma glucose | 2 | 1.8 | 2.21 | 0 |
| India | High body-mass index | 7.35 | 6.81 | 7.9 | 0 |
| India | Low physical activity | 0.9 | 0.64 | 1.15 | 0 |
| Niger | All cause | -0.16 | -0.25 | -0.07 | 0.001 |
| Niger | Tobacco | -0.25 | -0.41 | -0.1 | 0.002 |
| Niger | Alcohol use | 0.47 | 0.37 | 0.58 | 0 |
| Niger | High fasting plasma glucose | 1.19 | 1.08 | 1.3 | 0 |
| Niger | High body-mass index | 1.2 | 1.02 | 1.39 | 0 |
| Niger | Low physical activity | -0.04 | -0.12 | 0.04 | 0.354 |
| Nepal | All cause | 0.16 | -0.12 | 0.44 | 0.243 |
| Nepal | Tobacco | -0.14 | -0.22 | -0.05 | 0.003 |
| Nepal | Alcohol use | 3.52 | 2.94 | 4.11 | 0 |
| Nepal | High fasting plasma glucose | 0.88 | 0.55 | 1.21 | 0 |
| Nepal | High body-mass index | 5.42 | 5.16 | 5.68 | 0 |
| Nepal | Low physical activity | 0.22 | -0.06 | 0.51 | 0.123 |
| United Arab Emirates | All cause | 0.9 | 0.66 | 1.14 | 0 |
| United Arab Emirates | Tobacco | 1.46 | 1.23 | 1.69 | 0 |
| United Arab Emirates | Alcohol use | 0.75 | 0.5 | 1 | 0 |
| United Arab Emirates | High fasting plasma glucose | 1.89 | 1.47 | 2.3 | 0 |
| United Arab Emirates | High body-mass index | 3.03 | 2.61 | 3.45 | 0 |
| United Arab Emirates | Low physical activity | 0.99 | 0.73 | 1.25 | 0 |
| Bahrain | All cause | -1.59 | -1.97 | -1.2 | 0 |
| Bahrain | Tobacco | -2.72 | -3 | -2.44 | 0 |
| Bahrain | Alcohol use | -3.59 | -4.12 | -3.06 | 0 |
| Bahrain | High fasting plasma glucose | -0.62 | -1.11 | -0.13 | 0.015 |
| Bahrain | High body-mass index | -0.74 | -1.06 | -0.41 | 0 |
| Bahrain | Low physical activity | -1.53 | -1.9 | -1.15 | 0 |
| Azerbaijan | All cause | -0.13 | -0.43 | 0.16 | 0.364 |
| Azerbaijan | Tobacco | -0.24 | -0.51 | 0.04 | 0.089 |
| Azerbaijan | Alcohol use | 0.55 | 0.18 | 0.92 | 0.005 |
| Azerbaijan | High fasting plasma glucose | 0.7 | 0.23 | 1.17 | 0.005 |
| Azerbaijan | High body-mass index | 2.73 | 2 | 3.46 | 0 |
| Azerbaijan | Low physical activity | -0.09 | -0.38 | 0.21 | 0.552 |
| Bangladesh | All cause | 0.81 | 0.68 | 0.95 | 0 |
| Bangladesh | Tobacco | 0.98 | 0.82 | 1.14 | 0 |
| Bangladesh | Alcohol use | 2.85 | 2.62 | 3.07 | 0 |
| Bangladesh | High fasting plasma glucose | 2.23 | 2.11 | 2.35 | 0 |
| Bangladesh | High body-mass index | 9.41 | 8.65 | 10.18 | 0 |

| Bangladesh | Low physical activity | 0.96 | 0.8 | 1.13 | 0 |
| --- | --- | --- | --- | --- | --- |
| Mali | All cause | -0.09 | -0.18 | 0 | 0.045 |
| Mali | Tobacco | -0.38 | -0.48 | -0.28 | 0 |
| Mali | Alcohol use | -0.07 | -0.18 | 0.03 | 0.153 |
| Mali | High fasting plasma glucose | 1.16 | 1.12 | 1.21 | 0 |
| Mali | High body-mass index | 1.62 | 1.25 | 2 | 0 |
| Mali | Low physical activity | 0.08 | 0 | 0.16 | 0.041 |
| Philippines | All cause | 2.05 | 1.89 | 2.22 | 0 |
| Philippines | Tobacco | 2.21 | 1.93 | 2.49 | 0 |
| Philippines | Alcohol use | 1.9 | 1.7 | 2.1 | 0 |
| Philippines | High fasting plasma glucose | 3.69 | 3.39 | 3.98 | 0 |
| Philippines | High body-mass index | 4.12 | 3.83 | 4.42 | 0 |
| Philippines | Low physical activity | 2.11 | 1.95 | 2.28 | 0 |
| Uzbekistan | All cause | 1.34 | 1.11 | 1.56 | 0 |
| Uzbekistan | Tobacco | 0.99 | 0.7 | 1.28 | 0 |
| Uzbekistan | Alcohol use | 2.02 | 1.7 | 2.35 | 0 |
| Uzbekistan | High fasting plasma glucose | 2.66 | 2.26 | 3.06 | 0 |
| Uzbekistan | High body-mass index | 2.89 | 2.38 | 3.39 | 0 |
| Uzbekistan | Low physical activity | 1.45 | 1.23 | 1.67 | 0 |
| Panama | All cause | 0.24 | 0.02 | 0.47 | 0.034 |
| Panama | Tobacco | -1.16 | -1.38 | -0.93 | 0 |
| Panama | Alcohol use | 0.66 | 0.35 | 0.97 | 0 |
| Panama | High fasting plasma glucose | 0.71 | 0.48 | 0.93 | 0 |
| Panama | High body-mass index | 2.59 | 2.24 | 2.94 | 0 |
| Panama | Low physical activity | 0.29 | 0.06 | 0.51 | 0.014 |
| Middle SDI | All cause | 0.16 | 0.09 | 0.22 | 0 |
| Middle SDI | Tobacco | -0.59 | -0.72 | -0.46 | 0 |
| Middle SDI | Alcohol use | 0.51 | 0.37 | 0.65 | 0 |
| Middle SDI | High fasting plasma glucose | 1.1 | 1.04 | 1.16 | 0 |
| Middle SDI | High body-mass index | 3.19 | 3.1 | 3.27 | 0 |
| Middle SDI | Low physical activity | 0.26 | 0.19 | 0.33 | 0 |
| Pakistan | All cause | 1.32 | 0.99 | 1.65 | 0 |
| Pakistan | Tobacco | 0.97 | 0.55 | 1.39 | 0 |
| Pakistan | Alcohol use | -7.53 | -9.51 | -5.5 | 0 |
| Pakistan | High fasting plasma glucose | 3.24 | 2.97 | 3.51 | 0 |
| Pakistan | High body-mass index | 4.95 | 4.32 | 5.59 | 0 |
| Pakistan | Low physical activity | 1.43 | 1.12 | 1.74 | 0 |
| Seychelles | All cause | 1.05 | 0.89 | 1.21 | 0 |
| Seychelles | Tobacco | 0.92 | 0.78 | 1.07 | 0 |
| Seychelles | Alcohol use | 1.45 | 0.94 | 1.95 | 0 |
| Seychelles | High fasting plasma glucose | 2.49 | 2.24 | 2.74 | 0 |
| Seychelles | High body-mass index | 1.12 | 0.95 | 1.29 | 0 |
| Seychelles | Low physical activity | 1.21 | 1.03 | 1.4 | 0 |
| Haiti | All cause | 0.51 | 0.47 | 0.54 | 0 |
| Haiti | Tobacco | 0.21 | 0.12 | 0.3 | 0 |
| Haiti | Alcohol use | 0.49 | 0.4 | 0.57 | 0 |
| Haiti | High fasting plasma glucose | 0.65 | 0.58 | 0.71 | 0 |
| Haiti | High body-mass index | 3.44 | 3.18 | 3.71 | 0 |
| Haiti | Low physical activity | 0.54 | 0.51 | 0.58 | 0 |
| American Samoa | All cause | 1.42 | 1.29 | 1.54 | 0 |
| American Samoa | Tobacco | 1.39 | 1.28 | 1.51 | 0 |
| American Samoa | Alcohol use | 2.41 | 2.23 | 2.59 | 0 |
| American Samoa | High fasting plasma glucose | 2.2 | 2.08 | 2.33 | 0 |
| American Samoa | High body-mass index | 1.72 | 1.52 | 1.92 | 0 |
| American Samoa | Low physical activity | 1.35 | 1.23 | 1.48 | 0 |
| Egypt | All cause | 0.78 | 0.26 | 1.3 | 0.005 |
| Egypt | Tobacco | 0.6 | 0 | 1.21 | 0.049 |
| Egypt | Alcohol use | 0.95 | 0.34 | 1.56 | 0.003 |

| Egypt | High fasting plasma glucose | 2.37 | 1.91 | 2.83 | 0 |
| --- | --- | --- | --- | --- | --- |
| Egypt | High body-mass index | 2.7 | 1.85 | 3.55 | 0 |
| Egypt | Low physical activity | 0.84 | 0.33 | 1.35 | 0.002 |
| Nigeria | All cause | 0.15 | 0.09 | 0.22 | 0 |
| Nigeria | Tobacco | -1.22 | -1.34 | -1.1 | 0 |
| Nigeria | Alcohol use | -1.12 | -1.56 | -0.68 | 0 |
| Nigeria | High fasting plasma glucose | 1.15 | 1 | 1.29 | 0 |
| Nigeria | High body-mass index | 0.31 | -0.19 | 0.81 | 0.215 |
| Nigeria | Low physical activity | 0.27 | 0.21 | 0.33 | 0 |
| Albania | All cause | 1.47 | 1.13 | 1.81 | 0 |
| Albania | Tobacco | 1.41 | 1.19 | 1.63 | 0 |
| Albania | Alcohol use | 3.09 | 2.54 | 3.65 | 0 |
| Albania | High fasting plasma glucose | 2.38 | 2.01 | 2.75 | 0 |
| Albania | High body-mass index | 3.14 | 2.83 | 3.46 | 0 |
| Albania | Low physical activity | 1.63 | 1.29 | 1.98 | 0 |
| Sao Tome and Principe | All cause | 1.87 | 1.78 | 1.96 | 0 |
| Sao Tome and Principe | Tobacco | 1.52 | 1.42 | 1.62 | 0 |
| Sao Tome and Principe | Alcohol use | 2.19 | 2.01 | 2.36 | 0 |
| Sao Tome and Principe | High fasting plasma glucose | 3.38 | 3.31 | 3.44 | 0 |
| Sao Tome and Principe | High body-mass index | 2.99 | 2.75 | 3.23 | 0 |
| Sao Tome and Principe | Low physical activity | 2 | 1.92 | 2.08 | 0 |
| Timor-Leste | All cause | 0.7 | 0.48 | 0.92 | 0 |
| Timor-Leste | Tobacco | 0.05 | -0.14 | 0.24 | 0.599 |
| Timor-Leste | Alcohol use | -2.64 | -3.28 | -2 | 0 |
| Timor-Leste | High fasting plasma glucose | 1.23 | 1.01 | 1.45 | 0 |
| Timor-Leste | High body-mass index | 1.3 | 0.75 | 1.85 | 0 |
| Timor-Leste | Low physical activity | 0.73 | 0.52 | 0.94 | 0 |
| Central African Republic | All cause | 0.69 | 0.63 | 0.75 | 0 |
| Central African Republic | Tobacco | 1.14 | 1.01 | 1.27 | 0 |
| Central African Republic | Alcohol use | -0.01 | -0.13 | 0.11 | 0.88 |
| Central African Republic | High fasting plasma glucose | 1.46 | 1.4 | 1.52 | 0 |
| Central African Republic | High body-mass index | 2.54 | 2.31 | 2.77 | 0 |
| Central African Republic | Low physical activity | 0.66 | 0.6 | 0.71 | 0 |
| Vietnam | All cause | -0.22 | -0.26 | -0.17 | 0 |
| Vietnam | Tobacco | -1.03 | -1.21 | -0.84 | 0 |
| Vietnam | Alcohol use | 4.56 | 3.87 | 5.26 | 0 |
| Vietnam | High fasting plasma glucose | 0.72 | 0.64 | 0.81 | 0 |
| Vietnam | High body-mass index | 3.57 | 3.28 | 3.87 | 0 |
| Vietnam | Low physical activity | -0.09 | -0.13 | -0.05 | 0 |
| Mauritania | All cause | 0.1 | 0 | 0.19 | 0.046 |
| Mauritania | Tobacco | 0.26 | 0.15 | 0.36 | 0 |
| Mauritania | Alcohol use | -0.78 | -1.8 | 0.26 | 0.134 |
| Mauritania | High fasting plasma glucose | 1.37 | 1.28 | 1.46 | 0 |
| Mauritania | High body-mass index | 0.91 | 0.77 | 1.05 | 0 |
| Mauritania | Low physical activity | 0.3 | 0.22 | 0.38 | 0 |
| Algeria | All cause | 0.93 | 0.73 | 1.12 | 0 |
| Algeria | Tobacco | 0.85 | 0.64 | 1.06 | 0 |
| Algeria | Alcohol use | 1.53 | 1.12 | 1.95 | 0 |
| Algeria | High fasting plasma glucose | 2.14 | 1.91 | 2.37 | 0 |
| Algeria | High body-mass index | 2.81 | 2.49 | 3.13 | 0 |
| Algeria | Low physical activity | 0.97 | 0.78 | 1.16 | 0 |
| Bermuda | All cause | -2.68 | -2.87 | -2.49 | 0 |
| Bermuda | Tobacco | -2.79 | -2.99 | -2.59 | 0 |
| Bermuda | Alcohol use | -3.39 | -3.63 | -3.16 | 0 |
| Bermuda | High fasting plasma glucose | -2.54 | -2.75 | -2.33 | 0 |
| Bermuda | High body-mass index | -2.27 | -2.62 | -1.92 | 0 |
| Bermuda | Low physical activity | -2.56 | -2.76 | -2.37 | 0 |
| Lithuania | All cause | -0.27 | -0.56 | 0.02 | 0.067 |

| Lithuania | Tobacco | -1.21 | -1.49 | -0.94 | 0 |
| --- | --- | --- | --- | --- | --- |
| Lithuania | Alcohol use | 1.12 | 0.77 | 1.47 | 0 |
| Lithuania | High fasting plasma glucose | 0.55 | 0.26 | 0.83 | 0.001 |
| Lithuania | High body-mass index | 2.05 | 1.54 | 2.58 | 0 |
| Lithuania | Low physical activity | -0.01 | -0.31 | 0.29 | 0.946 |
| Somalia | All cause | 0.22 | 0.08 | 0.37 | 0.003 |
| Somalia | Tobacco | 0.09 | -0.04 | 0.22 | 0.178 |
| Somalia | Alcohol use | 2.26 | 1.88 | 2.63 | 0 |
| Somalia | High fasting plasma glucose | 0.99 | 0.87 | 1.11 | 0 |
| Somalia | High body-mass index | 2.46 | 2.15 | 2.77 | 0 |
| Somalia | Low physical activity | 0.22 | 0.09 | 0.36 | 0.003 |
| Senegal | All cause | 0.78 | 0.69 | 0.86 | 0 |
| Senegal | Tobacco | 0.19 | 0.05 | 0.34 | 0.009 |
| Senegal | Alcohol use | -0.35 | -0.5 | -0.21 | 0 |
| Senegal | High fasting plasma glucose | 2.21 | 2.09 | 2.34 | 0 |
| Senegal | High body-mass index | 1.71 | 1.58 | 1.83 | 0 |
| Senegal | Low physical activity | 0.81 | 0.72 | 0.9 | 0 |
| Greece | All cause | 0.05 | -0.18 | 0.28 | 0.649 |
| Greece | Tobacco | 0.58 | 0.45 | 0.71 | 0 |
| Greece | Alcohol use | -0.65 | -0.82 | -0.48 | 0 |
| Greece | High fasting plasma glucose | 1.41 | 1.06 | 1.76 | 0 |
| Greece | High body-mass index | 1.24 | 0.69 | 1.79 | 0 |
| Greece | Low physical activity | 0.24 | -0.02 | 0.51 | 0.07 |
| Tajikistan | All cause | 0.59 | 0.4 | 0.77 | 0 |
| Tajikistan | Tobacco | -0.53 | -0.87 | -0.19 | 0.003 |
| Tajikistan | Alcohol use | -0.79 | -1.01 | -0.56 | 0 |
| Tajikistan | High fasting plasma glucose | 1.4 | 1.12 | 1.67 | 0 |
| Tajikistan | High body-mass index | 0.13 | -0.44 | 0.71 | 0.644 |
| Tajikistan | Low physical activity | 0.64 | 0.43 | 0.86 | 0 |
| Moldova | All cause | -0.17 | -0.49 | 0.16 | 0.302 |
| Moldova | Tobacco | -0.75 | -1.07 | -0.44 | 0 |
| Moldova | Alcohol use | -1.59 | -1.94 | -1.25 | 0 |
| Moldova | High fasting plasma glucose | 0.51 | 0.17 | 0.85 | 0.004 |
| Moldova | High body-mass index | 3.77 | 3.21 | 4.34 | 0 |
| Moldova | Low physical activity | 0.16 | -0.17 | 0.5 | 0.324 |
| Virgin Islands, U.S. | All cause | 0.16 | 0.04 | 0.27 | 0.009 |
| Virgin Islands, U.S. | Tobacco | 0.45 | 0.36 | 0.54 | 0 |
| Virgin Islands, U.S. | Alcohol use | 0.31 | 0.22 | 0.39 | 0 |
| Virgin Islands, U.S. | High fasting plasma glucose | 0.51 | 0.36 | 0.66 | 0 |
| Virgin Islands, U.S. | High body-mass index | 0.19 | 0.02 | 0.36 | 0.033 |
| Virgin Islands, U.S. | Low physical activity | 0.15 | 0.03 | 0.27 | 0.017 |
| Tanzania | All cause | -0.33 | -0.54 | -0.13 | 0.003 |
| Tanzania | Tobacco | -0.2 | -0.41 | 0.01 | 0.056 |
| Tanzania | Alcohol use | -1.07 | -1.24 | -0.89 | 0 |
| Tanzania | High fasting plasma glucose | 0.77 | 0.58 | 0.96 | 0 |
| Tanzania | High body-mass index | 1.3 | 1.13 | 1.47 | 0 |
| Tanzania | Low physical activity | -0.29 | -0.48 | -0.09 | 0.006 |
| Congo | All cause | 0.65 | 0.5 | 0.8 | 0 |
| Congo | Tobacco | 1.03 | 0.89 | 1.18 | 0 |
| Congo | Alcohol use | 0.46 | 0.24 | 0.67 | 0 |
| Congo | High fasting plasma glucose | 1.48 | 1.34 | 1.62 | 0 |
| Congo | High body-mass index | 2.5 | 2.34 | 2.65 | 0 |
| Congo | Low physical activity | 0.73 | 0.58 | 0.87 | 0 |
| Jamaica | All cause | 0.97 | 0.62 | 1.31 | 0 |
| Jamaica | Tobacco | 0.52 | 0.15 | 0.89 | 0.007 |
| Jamaica | Alcohol use | 1.45 | 1.11 | 1.8 | 0 |
| Jamaica | High fasting plasma glucose | 1.75 | 1.32 | 2.18 | 0 |
| Jamaica | High body-mass index | 1.98 | 1.64 | 2.32 | 0 |

| Jamaica | Low physical activity | 0.94 | 0.59 | 1.29 | 0 |
| --- | --- | --- | --- | --- | --- |
| Cyprus | All cause | -0.94 | -1.23 | -0.66 | 0 |
| Cyprus | Tobacco | -1.12 | -1.56 | -0.69 | 0 |
| Cyprus | Alcohol use | -1.6 | -1.86 | -1.33 | 0 |
| Cyprus | High fasting plasma glucose | -0.8 | -1.18 | -0.43 | 0 |
| Cyprus | High body-mass index | 0.9 | 0.53 | 1.28 | 0 |
| Cyprus | Low physical activity | -0.85 | -1.14 | -0.57 | 0 |
| Brunei | All cause | 1.34 | 1.05 | 1.62 | 0 |
| Brunei | Tobacco | 0.11 | -0.2 | 0.42 | 0.464 |
| Brunei | Alcohol use | -0.77 | -2.04 | 0.52 | 0.229 |
| Brunei | High fasting plasma glucose | 1.19 | 0.86 | 1.51 | 0 |
| Brunei | High body-mass index | 2.57 | 2.2 | 2.94 | 0 |
| Brunei | Low physical activity | 1.28 | 0.98 | 1.59 | 0 |
| Iran | All cause | 1.76 | 1.36 | 2.17 | 0 |
| Iran | Tobacco | 1.99 | 1.58 | 2.4 | 0 |
| Iran | Alcohol use | -26.98 | -32.15 | -21.41 | 0 |
| Iran | High fasting plasma glucose | 3.6 | 2.97 | 4.23 | 0 |
| Iran | High body-mass index | 3.87 | 3.44 | 4.31 | 0 |
| Iran | Low physical activity | 1.82 | 1.41 | 2.24 | 0 |
| Bosnia and Herzegovina | All cause | 1.77 | 1.56 | 1.97 | 0 |
| Bosnia and Herzegovina | Tobacco | 2.06 | 1.84 | 2.28 | 0 |
| Bosnia and Herzegovina | Alcohol use | 0.96 | 0.8 | 1.12 | 0 |
| Bosnia and Herzegovina | High fasting plasma glucose | 4.13 | 3.79 | 4.48 | 0 |
| Bosnia and Herzegovina | High body-mass index | 5.18 | 4.68 | 5.67 | 0 |
| Bosnia and Herzegovina | Low physical activity | 2.18 | 1.96 | 2.4 | 0 |
| Iraq | All cause | -2.39 | -2.85 | -1.94 | 0 |
| Iraq | Tobacco | -2.85 | -3.24 | -2.46 | 0 |
| Iraq | Alcohol use | -4.6 | -4.94 | -4.26 | 0 |
| Iraq | High fasting plasma glucose | -2.5 | -2.98 | -2.02 | 0 |
| Iraq | High body-mass index | 0.56 | -0.14 | 1.27 | 0.112 |
| Iraq | Low physical activity | -2.44 | -2.88 | -2 | 0 |
| Sri Lanka | All cause | 0.42 | 0.3 | 0.55 | 0 |
| Sri Lanka | Tobacco | 0.3 | 0.19 | 0.41 | 0 |
| Sri Lanka | Alcohol use | 4.77 | 4.5 | 5.04 | 0 |
| Sri Lanka | High fasting plasma glucose | 2.67 | 2.5 | 2.84 | 0 |
| Sri Lanka | High body-mass index | 2.91 | 2.73 | 3.1 | 0 |
| Sri Lanka | Low physical activity | 0.58 | 0.44 | 0.72 | 0 |
| High-middle SDI | All cause | -0.49 | -0.65 | -0.33 | 0 |
| High-middle SDI | Tobacco | -1.01 | -1.2 | -0.83 | 0 |
| High-middle SDI | Alcohol use | -1.07 | -1.34 | -0.79 | 0 |
| High-middle SDI | High fasting plasma glucose | 0.19 | -0.02 | 0.4 | 0.07 |
| High-middle SDI | High body-mass index | 2.33 | 2.18 | 2.48 | 0 |
| High-middle SDI | Low physical activity | -0.34 | -0.48 | -0.2 | 0 |
| Turkmenistan | All cause | 1.59 | 1.23 | 1.95 | 0 |
| Turkmenistan | Tobacco | 1.56 | 1.19 | 1.93 | 0 |
| Turkmenistan | Alcohol use | 2.23 | 1.88 | 2.59 | 0 |
| Turkmenistan | High fasting plasma glucose | 2.54 | 2.27 | 2.81 | 0 |
| Turkmenistan | High body-mass index | 1.1 | 0.7 | 1.49 | 0 |
| Turkmenistan | Low physical activity | 1.78 | 1.46 | 2.11 | 0 |
| Russian Federation | All cause | -0.05 | -0.53 | 0.43 | 0.829 |
| Russian Federation | Tobacco | -0.18 | -0.59 | 0.24 | 0.387 |
| Russian Federation | Alcohol use | 1.17 | 0.55 | 1.78 | 0.001 |
| Russian Federation | High fasting plasma glucose | 0.61 | 0.15 | 1.07 | 0.011 |
| Russian Federation | High body-mass index | 2.44 | 1.84 | 3.05 | 0 |
| Russian Federation | Low physical activity | 0.14 | -0.27 | 0.56 | 0.487 |
| Saint Vincent and the Gren | All cause | 0.2 | -0.04 | 0.43 | 0.1 |
| Saint Vincent and the Gren | Tobacco | 0.11 | -0.15 | 0.37 | 0.395 |
| Saint Vincent and the Gren | Alcohol use | 0.73 | 0.43 | 1.03 | 0 |

| Saint Vincent and the Gren | High fasting plasma glucose | 0.46 | 0.21 | 0.7 | 0.001 |
| --- | --- | --- | --- | --- | --- |
| Saint Vincent and the Gren | High body-mass index | 2.46 | 2.21 | 2.7 | 0 |
| Saint Vincent and the Gren | Low physical activity | 0.26 | 0.02 | 0.49 | 0.032 |
| Fiji | All cause | 0.64 | 0.45 | 0.84 | 0 |
| Fiji | Tobacco | 0.23 | 0.04 | 0.42 | 0.018 |
| Fiji | Alcohol use | 1.79 | 1.4 | 2.18 | 0 |
| Fiji | High fasting plasma glucose | 1.4 | 1.27 | 1.54 | 0 |
| Fiji | High body-mass index | 1.21 | 1.12 | 1.29 | 0 |
| Fiji | Low physical activity | 0.66 | 0.46 | 0.86 | 0 |
| Democratic Republic of the | All cause | 0.57 | 0.43 | 0.71 | 0 |
| Democratic Republic of the | Tobacco | 0.22 | 0.12 | 0.32 | 0 |
| Democratic Republic of the | Alcohol use | 0.95 | 0.54 | 1.36 | 0 |
| Democratic Republic of the | High fasting plasma glucose | 1.21 | 1.04 | 1.39 | 0 |
| Democratic Republic of the | High body-mass index | 0.33 | -0.13 | 0.79 | 0.151 |
| Democratic Republic of the | Low physical activity | 0.56 | 0.41 | 0.71 | 0 |
| Comoros | All cause | 0.4 | 0.27 | 0.53 | 0 |
| Comoros | Tobacco | -0.09 | -0.21 | 0.03 | 0.146 |
| Comoros | Alcohol use | 0.98 | 0.76 | 1.19 | 0 |
| Comoros | High fasting plasma glucose | 0.95 | 0.82 | 1.07 | 0 |
| Comoros | High body-mass index | 2.17 | 1.89 | 2.46 | 0 |
| Comoros | Low physical activity | 0.47 | 0.35 | 0.59 | 0 |
| Botswana | All cause | 1.88 | 1.4 | 2.37 | 0 |
| Botswana | Tobacco | 1.83 | 1.35 | 2.31 | 0 |
| Botswana | Alcohol use | 2.49 | 1.93 | 3.06 | 0 |
| Botswana | High fasting plasma glucose | 3.13 | 2.57 | 3.69 | 0 |
| Botswana | High body-mass index | 3.42 | 3.05 | 3.79 | 0 |
| Botswana | Low physical activity | 1.91 | 1.45 | 2.38 | 0 |
| Sierra Leone | All cause | 1.42 | 1.36 | 1.49 | 0 |
| Sierra Leone | Tobacco | 1.08 | 0.97 | 1.2 | 0 |
| Sierra Leone | Alcohol use | 1.42 | 1.29 | 1.55 | 0 |
| Sierra Leone | High fasting plasma glucose | 3.06 | 2.9 | 3.22 | 0 |
| Sierra Leone | High body-mass index | 3.71 | 3.5 | 3.93 | 0 |
| Sierra Leone | Low physical activity | 1.5 | 1.44 | 1.56 | 0 |
| Honduras | All cause | 0.39 | 0.17 | 0.61 | 0.001 |
| Honduras | Tobacco | -0.06 | -0.35 | 0.23 | 0.669 |
| Honduras | Alcohol use | 0.55 | 0.36 | 0.74 | 0 |
| Honduras | High fasting plasma glucose | 1.42 | 1.15 | 1.7 | 0 |
| Honduras | High body-mass index | 2.28 | 1.62 | 2.94 | 0 |
| Honduras | Low physical activity | 0.61 | 0.39 | 0.83 | 0 |
| Bulgaria | All cause | 0.35 | 0.23 | 0.47 | 0 |
| Bulgaria | Tobacco | -0.07 | -0.18 | 0.05 | 0.224 |
| Bulgaria | Alcohol use | -0.26 | -0.47 | -0.05 | 0.016 |
| Bulgaria | High fasting plasma glucose | 0.74 | 0.54 | 0.94 | 0 |
| Bulgaria | High body-mass index | 1.96 | 1.76 | 2.15 | 0 |
| Bulgaria | Low physical activity | 0.47 | 0.35 | 0.59 | 0 |
| Djibouti | All cause | 0.45 | 0.4 | 0.51 | 0 |
| Djibouti | Tobacco | 1.36 | 1.19 | 1.52 | 0 |
| Djibouti | Alcohol use | 0.78 | 0.46 | 1.09 | 0 |
| Djibouti | High fasting plasma glucose | 1.46 | 1.4 | 1.52 | 0 |
| Djibouti | High body-mass index | 5.92 | 5.69 | 6.14 | 0 |
| Djibouti | Low physical activity | 0.63 | 0.58 | 0.69 | 0 |
| Saint Lucia | All cause | -0.38 | -0.67 | -0.08 | 0.015 |
| Saint Lucia | Tobacco | -0.3 | -0.56 | -0.04 | 0.028 |
| Saint Lucia | Alcohol use | -1.27 | -1.5 | -1.04 | 0 |
| Saint Lucia | High fasting plasma glucose | -0.54 | -0.84 | -0.22 | 0.002 |
| Saint Lucia | High body-mass index | 1.63 | 1.36 | 1.89 | 0 |
| Saint Lucia | Low physical activity | -0.31 | -0.6 | -0.02 | 0.034 |
| Thailand | All cause | 0.15 | -0.19 | 0.5 | 0.364 |

| Thailand | Tobacco | -1.05 | -1.45 | -0.65 | 0 |
| --- | --- | --- | --- | --- | --- |
| Thailand | Alcohol use | 0.7 | 0.25 | 1.15 | 0.003 |
| Thailand | High fasting plasma glucose | 0.32 | 0.04 | 0.61 | 0.026 |
| Thailand | High body-mass index | 4.01 | 3.5 | 4.51 | 0 |
| Thailand | Low physical activity | 0.24 | -0.09 | 0.57 | 0.15 |
| Equatorial Guinea | All cause | 0.52 | 0.33 | 0.7 | 0 |
| Equatorial Guinea | Tobacco | 0.34 | 0.16 | 0.51 | 0 |
| Equatorial Guinea | Alcohol use | 2.42 | 2.16 | 2.68 | 0 |
| Equatorial Guinea | High fasting plasma glucose | 1.7 | 1.51 | 1.89 | 0 |
| Equatorial Guinea | High body-mass index | 6.88 | 6.28 | 7.48 | 0 |
| Equatorial Guinea | Low physical activity | 0.85 | 0.67 | 1.04 | 0 |
| Suriname | All cause | 0.56 | 0.4 | 0.72 | 0 |
| Suriname | Tobacco | 0.91 | 0.73 | 1.09 | 0 |
| Suriname | Alcohol use | 2.03 | 1.74 | 2.31 | 0 |
| Suriname | High fasting plasma glucose | 1.61 | 1.47 | 1.76 | 0 |
| Suriname | High body-mass index | 2.25 | 1.9 | 2.6 | 0 |
| Suriname | Low physical activity | 0.61 | 0.45 | 0.76 | 0 |
| Lesotho | All cause | 2.44 | 1.9 | 2.99 | 0 |
| Lesotho | Tobacco | 2.93 | 2.34 | 3.51 | 0 |
| Lesotho | Alcohol use | 2.73 | 2.13 | 3.34 | 0 |
| Lesotho | High fasting plasma glucose | 3.58 | 2.96 | 4.21 | 0 |
| Lesotho | High body-mass index | 4.43 | 3.89 | 4.97 | 0 |
| Lesotho | Low physical activity | 2.53 | 1.97 | 3.08 | 0 |
| Papua New Guinea | All cause | 0.45 | 0.38 | 0.52 | 0 |
| Papua New Guinea | Tobacco | 0.49 | 0.39 | 0.58 | 0 |
| Papua New Guinea | Alcohol use | 0.24 | 0.18 | 0.31 | 0 |
| Papua New Guinea | High fasting plasma glucose | 1.12 | 1.07 | 1.17 | 0 |
| Papua New Guinea | High body-mass index | 1.04 | 0.75 | 1.33 | 0 |
| Papua New Guinea | Low physical activity | 0.49 | 0.42 | 0.56 | 0 |
| Ukraine | All cause | -0.68 | -1.04 | -0.32 | 0.001 |
| Ukraine | Tobacco | -1.33 | -1.72 | -0.95 | 0 |
| Ukraine | Alcohol use | -0.21 | -0.63 | 0.22 | 0.327 |
| Ukraine | High fasting plasma glucose | -0.22 | -0.62 | 0.18 | 0.268 |
| Ukraine | High body-mass index | 1.73 | 1.23 | 2.22 | 0 |
| Ukraine | Low physical activity | -0.48 | -0.76 | -0.2 | 0.002 |
| Uganda | All cause | -0.74 | -0.94 | -0.55 | 0 |
| Uganda | Tobacco | -0.88 | -1.1 | -0.67 | 0 |
| Uganda | Alcohol use | -1.24 | -1.56 | -0.93 | 0 |
| Uganda | High fasting plasma glucose | -0.33 | -0.54 | -0.11 | 0.004 |
| Uganda | High body-mass index | 3.86 | 3.63 | 4.08 | 0 |
| Uganda | Low physical activity | -0.67 | -0.86 | -0.49 | 0 |
| Kiribati | All cause | 0.39 | 0.23 | 0.55 | 0 |
| Kiribati | Tobacco | 1.45 | 1.28 | 1.63 | 0 |
| Kiribati | Alcohol use | 0.64 | 0.45 | 0.83 | 0 |
| Kiribati | High fasting plasma glucose | 1.1 | 0.97 | 1.22 | 0 |
| Kiribati | High body-mass index | 1.05 | 0.82 | 1.28 | 0 |
| Kiribati | Low physical activity | 0.38 | 0.22 | 0.54 | 0 |
| Bolivia | All cause | 0.14 | -0.06 | 0.35 | 0.168 |
| Bolivia | Tobacco | -0.11 | -0.29 | 0.06 | 0.204 |
| Bolivia | Alcohol use | 0.05 | -0.04 | 0.13 | 0.29 |
| Bolivia | High fasting plasma glucose | 0.56 | 0.36 | 0.76 | 0 |
| Bolivia | High body-mass index | 3.3 | 2.94 | 3.66 | 0 |
| Bolivia | Low physical activity | 0.23 | 0.03 | 0.44 | 0.029 |
| Macedonia | All cause | -0.14 | -0.28 | 0.01 | 0.061 |
| Macedonia | Tobacco | 0.03 | -0.15 | 0.21 | 0.73 |
| Macedonia | Alcohol use | -2.04 | -2.38 | -1.71 | 0 |
| Macedonia | High fasting plasma glucose | 0.96 | 0.69 | 1.23 | 0 |
| Macedonia | High body-mass index | 1.1 | 0.76 | 1.44 | 0 |

| Macedonia | Low physical activity | 0.07 | -0.08 | 0.22 | 0.354 |
| --- | --- | --- | --- | --- | --- |
| Antigua and Barbuda | All cause | 0.64 | 0.54 | 0.74 | 0 |
| Antigua and Barbuda | Tobacco | 0.36 | 0.25 | 0.47 | 0 |
| Antigua and Barbuda | Alcohol use | 3.41 | 3.09 | 3.73 | 0 |
| Antigua and Barbuda | High fasting plasma glucose | 1.25 | 1.13 | 1.38 | 0 |
| Antigua and Barbuda | High body-mass index | 2.64 | 2.37 | 2.91 | 0 |
| Antigua and Barbuda | Low physical activity | 0.75 | 0.66 | 0.85 | 0 |
| Hungary | All cause | -1.48 | -1.72 | -1.25 | 0 |
| Hungary | Tobacco | -1.7 | -1.93 | -1.47 | 0 |
| Hungary | Alcohol use | -2.38 | -2.65 | -2.11 | 0 |
| Hungary | High fasting plasma glucose | -1.01 | -1.23 | -0.78 | 0 |
| Hungary | High body-mass index | -0.14 | -0.46 | 0.19 | 0.389 |
| Hungary | Low physical activity | -1.38 | -1.62 | -1.15 | 0 |
| Morocco | All cause | 0.48 | 0.39 | 0.56 | 0 |
| Morocco | Tobacco | -0.03 | -0.16 | 0.1 | 0.617 |
| Morocco | Alcohol use | 0.07 | -0.11 | 0.26 | 0.43 |
| Morocco | High fasting plasma glucose | 1.5 | 1.38 | 1.62 | 0 |
| Morocco | High body-mass index | 1.76 | 1.27 | 2.25 | 0 |
| Morocco | Low physical activity | 0.49 | 0.41 | 0.56 | 0 |
| Zambia | All cause | -1.22 | -1.69 | -0.75 | 0 |
| Zambia | Tobacco | -0.73 | -1.15 | -0.3 | 0.002 |
| Zambia | Alcohol use | -1.95 | -2.48 | -1.41 | 0 |
| Zambia | High fasting plasma glucose | -0.92 | -1.43 | -0.4 | 0.001 |
| Zambia | High body-mass index | 2.53 | 1.81 | 3.26 | 0 |
| Zambia | Low physical activity | -1.1 | -1.55 | -0.64 | 0 |
| Australia | All cause | -1.56 | -1.71 | -1.42 | 0 |
| Australia | Tobacco | -3.01 | -3.24 | -2.77 | 0 |
| Australia | Alcohol use | -1.92 | -2.11 | -1.72 | 0 |
| Australia | High fasting plasma glucose | -1.34 | -1.55 | -1.13 | 0 |
| Australia | High body-mass index | 0.1 | -0.04 | 0.25 | 0.146 |
| Australia | Low physical activity | -1.42 | -1.56 | -1.28 | 0 |
| Japan | All cause | 0.89 | 0.65 | 1.15 | 0 |
| Japan | Tobacco | 0 | -0.2 | 0.21 | 0.973 |
| Japan | Alcohol use | 0.52 | 0.29 | 0.75 | 0 |
| Japan | High fasting plasma glucose | 1.23 | 1.01 | 1.45 | 0 |
| Japan | High body-mass index | 2.83 | 2.51 | 3.14 | 0 |
| Japan | Low physical activity | 0.87 | 0.65 | 1.08 | 0 |
| Samoa | All cause | -0.48 | -0.59 | -0.37 | 0 |
| Samoa | Tobacco | -0.28 | -0.36 | -0.2 | 0 |
| Samoa | Alcohol use | 0.17 | 0.03 | 0.31 | 0.017 |
| Samoa | High fasting plasma glucose | 1.03 | 0.92 | 1.14 | 0 |
| Samoa | High body-mass index | 0.24 | 0.17 | 0.31 | 0 |
| Samoa | Low physical activity | -0.45 | -0.56 | -0.33 | 0 |
| Ecuador | All cause | 1.06 | 0.85 | 1.28 | 0 |
| Ecuador | Tobacco | 0.83 | 0.64 | 1.02 | 0 |
| Ecuador | Alcohol use | 1.57 | 1.27 | 1.87 | 0 |
| Ecuador | High fasting plasma glucose | 1.95 | 1.73 | 2.16 | 0 |
| Ecuador | High body-mass index | 3.91 | 3.6 | 4.21 | 0 |
| Ecuador | Low physical activity | 1.11 | 0.91 | 1.31 | 0 |
| The Bahamas | All cause | 0.82 | 0.7 | 0.94 | 0 |
| The Bahamas | Tobacco | 0.49 | 0.37 | 0.6 | 0 |
| The Bahamas | Alcohol use | -0.52 | -0.71 | -0.33 | 0 |
| The Bahamas | High fasting plasma glucose | 1.05 | 0.92 | 1.17 | 0 |
| The Bahamas | High body-mass index | 1.54 | 1.07 | 2.02 | 0 |
| The Bahamas | Low physical activity | 0.85 | 0.73 | 0.96 | 0 |
| Luxembourg | All cause | -1.34 | -1.46 | -1.22 | 0 |
| Luxembourg | Tobacco | -2.23 | -2.33 | -2.13 | 0 |
| Luxembourg | Alcohol use | -0.29 | -0.47 | -0.11 | 0.003 |

| Luxembourg | High fasting plasma glucose | -0.37 | -0.51 | -0.24 | 0 |
| --- | --- | --- | --- | --- | --- |
| Luxembourg | High body-mass index | 0.01 | -0.1 | 0.13 | 0.82 |
| Luxembourg | Low physical activity | -1.21 | -1.33 | -1.09 | 0 |
| Montenegro | All cause | 0.11 | -0.09 | 0.31 | 0.267 |
| Montenegro | Tobacco | 0.98 | 0.74 | 1.21 | 0 |
| Montenegro | Alcohol use | -0.34 | -0.46 | -0.22 | 0 |
| Montenegro | High fasting plasma glucose | 0.76 | 0.58 | 0.94 | 0 |
| Montenegro | High body-mass index | 0.93 | 0.84 | 1.01 | 0 |
| Montenegro | Low physical activity | 0.17 | 0.01 | 0.34 | 0.041 |
| New Zealand | All cause | -1.57 | -1.66 | -1.48 | 0 |
| New Zealand | Tobacco | -3.06 | -3.17 | -2.96 | 0 |
| New Zealand | Alcohol use | -1.66 | -1.85 | -1.47 | 0 |
| New Zealand | High fasting plasma glucose | -1.95 | -2.27 | -1.63 | 0 |
| New Zealand | High body-mass index | -0.76 | -0.99 | -0.53 | 0 |
| New Zealand | Low physical activity | -1.6 | -1.7 | -1.51 | 0 |
| Palestine | All cause | 0.7 | 0.55 | 0.86 | 0 |
| Palestine | Tobacco | 0.21 | 0.1 | 0.32 | 0.001 |
| Palestine | Alcohol use | 0.05 | -1.38 | 1.5 | 0.949 |
| Palestine | High fasting plasma glucose | 1.75 | 1.57 | 1.93 | 0 |
| Palestine | High body-mass index | 3.63 | 3.28 | 3.97 | 0 |
| Palestine | Low physical activity | 0.79 | 0.62 | 0.95 | 0 |
| Togo | All cause | 0.28 | 0.16 | 0.41 | 0 |
| Togo | Tobacco | 0.12 | -0.04 | 0.28 | 0.134 |
| Togo | Alcohol use | -0.66 | -0.97 | -0.34 | 0 |
| Togo | High fasting plasma glucose | 1.6 | 1.47 | 1.73 | 0 |
| Togo | High body-mass index | 1.61 | 1.28 | 1.94 | 0 |
| Togo | Low physical activity | 0.43 | 0.31 | 0.54 | 0 |
| Angola | All cause | 0.32 | 0.25 | 0.39 | 0 |
| Angola | Tobacco | 0.94 | 0.85 | 1.03 | 0 |
| Angola | Alcohol use | 4.29 | 3.93 | 4.66 | 0 |
| Angola | High fasting plasma glucose | 1.13 | 1.08 | 1.19 | 0 |
| Angola | High body-mass index | 4.73 | 4.65 | 4.82 | 0 |
| Angola | Low physical activity | 0.47 | 0.4 | 0.53 | 0 |
| Netherlands | All cause | -1.71 | -1.89 | -1.53 | 0 |
| Netherlands | Tobacco | -2.79 | -3.04 | -2.54 | 0 |
| Netherlands | Alcohol use | -1.92 | -2.13 | -1.72 | 0 |
| Netherlands | High fasting plasma glucose | -1.15 | -1.31 | -0.98 | 0 |
| Netherlands | High body-mass index | -1.19 | -1.36 | -1.01 | 0 |
| Netherlands | Low physical activity | -1.61 | -1.78 | -1.44 | 0 |
| Namibia | All cause | 0.82 | 0.34 | 1.3 | 0.001 |
| Namibia | Tobacco | 0.61 | 0.12 | 1.09 | 0.017 |
| Namibia | Alcohol use | 7.13 | 5.28 | 9.01 | 0 |
| Namibia | High fasting plasma glucose | 1.33 | 0.78 | 1.88 | 0 |
| Namibia | High body-mass index | 2.68 | 2.36 | 3.01 | 0 |
| Namibia | Low physical activity | 0.97 | 0.48 | 1.46 | 0 |
| Croatia | All cause | -0.08 | -0.27 | 0.1 | 0.364 |
| Croatia | Tobacco | -1.02 | -1.26 | -0.78 | 0 |
| Croatia | Alcohol use | -0.84 | -1.18 | -0.49 | 0 |
| Croatia | High fasting plasma glucose | 0.94 | 0.74 | 1.13 | 0 |
| Croatia | High body-mass index | 1.73 | 1.47 | 2 | 0 |
| Croatia | Low physical activity | 0.25 | 0.08 | 0.43 | 0.006 |
| Malta | All cause | -1.28 | -1.37 | -1.19 | 0 |
| Malta | Tobacco | -2.32 | -2.49 | -2.14 | 0 |
| Malta | Alcohol use | -0.99 | -1.17 | -0.8 | 0 |
| Malta | High fasting plasma glucose | -0.68 | -0.78 | -0.57 | 0 |
| Malta | High body-mass index | -0.31 | -0.48 | -0.13 | 0.002 |
| Malta | Low physical activity | -1.22 | -1.31 | -1.13 | 0 |
| South Korea | All cause | 0.83 | 0.65 | 1.02 | 0 |

| South Korea | Tobacco | -0.82 | -1.03 | -0.61 | 0 |
| --- | --- | --- | --- | --- | --- |
| South Korea | Alcohol use | 0.56 | 0.34 | 0.78 | 0 |
| South Korea | High fasting plasma glucose | 1.46 | 1.25 | 1.67 | 0 |
| South Korea | High body-mass index | 2.34 | 1.96 | 2.72 | 0 |
| South Korea | Low physical activity | 0.86 | 0.71 | 1.02 | 0 |
| Iceland | All cause | -2.17 | -2.28 | -2.05 | 0 |
| Iceland | Tobacco | -3.45 | -3.62 | -3.28 | 0 |
| Iceland | Alcohol use | -1.22 | -1.32 | -1.11 | 0 |
| Iceland | High fasting plasma glucose | -0.86 | -0.98 | -0.73 | 0 |
| Iceland | High body-mass index | -1.08 | -1.27 | -0.89 | 0 |
| Iceland | Low physical activity | -2.06 | -2.17 | -1.95 | 0 |
| Czech Republic | All cause | -1.69 | -1.94 | -1.44 | 0 |
| Czech Republic | Tobacco | -2.34 | -2.61 | -2.07 | 0 |
| Czech Republic | Alcohol use | -1.66 | -2.04 | -1.29 | 0 |
| Czech Republic | High fasting plasma glucose | -0.75 | -0.91 | -0.6 | 0 |
| Czech Republic | High body-mass index | -0.3 | -0.76 | 0.16 | 0.187 |
| Czech Republic | Low physical activity | -1.5 | -1.77 | -1.24 | 0 |
| Poland | All cause | -0.39 | -0.49 | -0.29 | 0 |
| Poland | Tobacco | -1.03 | -1.14 | -0.93 | 0 |
| Poland | Alcohol use | -0.05 | -0.15 | 0.05 | 0.31 |
| Poland | High fasting plasma glucose | 0.3 | 0.21 | 0.4 | 0 |
| Poland | High body-mass index | 1.47 | 1.3 | 1.64 | 0 |
| Poland | Low physical activity | -0.18 | -0.28 | -0.07 | 0.002 |
| Gabon | All cause | 0.5 | 0.26 | 0.75 | 0 |
| Gabon | Tobacco | 0.58 | 0.28 | 0.89 | 0 |
| Gabon | Alcohol use | 0.07 | -0.05 | 0.2 | 0.247 |
| Gabon | High fasting plasma glucose | 1.56 | 1.24 | 1.88 | 0 |
| Gabon | High body-mass index | 2.54 | 2.09 | 3 | 0 |
| Gabon | Low physical activity | 0.64 | 0.4 | 0.88 | 0 |
| Benin | All cause | 0.64 | 0.56 | 0.72 | 0 |
| Benin | Tobacco | -0.05 | -0.14 | 0.05 | 0.308 |
| Benin | Alcohol use | 0.93 | 0.85 | 1.02 | 0 |
| Benin | High fasting plasma glucose | 2.22 | 2.14 | 2.29 | 0 |
| Benin | High body-mass index | 2.47 | 2.25 | 2.69 | 0 |
| Benin | Low physical activity | 0.85 | 0.78 | 0.92 | 0 |
| South Africa | All cause | 0.15 | -0.36 | 0.67 | 0.551 |
| South Africa | Tobacco | -1.37 | -1.83 | -0.92 | 0 |
| South Africa | Alcohol use | -0.1 | -0.62 | 0.41 | 0.685 |
| South Africa | High fasting plasma glucose | 1.51 | 1.02 | 2.01 | 0 |
| South Africa | High body-mass index | 3.02 | 2.46 | 3.57 | 0 |
| South Africa | Low physical activity | 0.24 | -0.22 | 0.69 | 0.293 |
| Peru | All cause | -0.18 | -0.39 | 0.03 | 0.083 |
| Peru | Tobacco | -0.53 | -0.77 | -0.29 | 0 |
| Peru | Alcohol use | -0.83 | -1.04 | -0.62 | 0 |
| Peru | High fasting plasma glucose | 0.58 | 0.4 | 0.76 | 0 |
| Peru | High body-mass index | 2.84 | 2.62 | 3.06 | 0 |
| Peru | Low physical activity | -0.08 | -0.29 | 0.12 | 0.404 |
| Lebanon | All cause | 0.45 | 0.38 | 0.52 | 0 |
| Lebanon | Tobacco | 0.81 | 0.58 | 1.04 | 0 |
| Lebanon | Alcohol use | -0.77 | -1.14 | -0.4 | 0 |
| Lebanon | High fasting plasma glucose | 1.22 | 1.09 | 1.34 | 0 |
| Lebanon | High body-mass index | 2.99 | 2.9 | 3.07 | 0 |
| Lebanon | Low physical activity | 0.61 | 0.55 | 0.68 | 0 |
| Kenya | All cause | 0.25 | 0.11 | 0.39 | 0.001 |
| Kenya | Tobacco | 0.49 | 0.33 | 0.64 | 0 |
| Kenya | Alcohol use | 0.1 | -0.02 | 0.22 | 0.087 |
| Kenya | High fasting plasma glucose | 0.78 | 0.66 | 0.91 | 0 |
| Kenya | High body-mass index | 3.49 | 2.49 | 4.5 | 0 |

| Kenya | Low physical activity | 0.22 | 0.08 | 0.35 | 0.003 |
| --- | --- | --- | --- | --- | --- |
| Ireland | All cause | -1.67 | -1.77 | -1.57 | 0 |
| Ireland | Tobacco | -3.12 | -3.26 | -2.98 | 0 |
| Ireland | Alcohol use | -1.68 | -1.89 | -1.46 | 0 |
| Ireland | High fasting plasma glucose | -0.63 | -0.74 | -0.51 | 0 |
| Ireland | High body-mass index | -0.25 | -0.39 | -0.11 | 0.001 |
| Ireland | Low physical activity | -1.57 | -1.67 | -1.47 | 0 |
| Burkina Faso | All cause | -0.27 | -0.4 | -0.13 | 0 |
| Burkina Faso | Tobacco | -0.58 | -0.65 | -0.5 | 0 |
| Burkina Faso | Alcohol use | -0.62 | -0.71 | -0.54 | 0 |
| Burkina Faso | High fasting plasma glucose | 0.89 | 0.79 | 0.98 | 0 |
| Burkina Faso | High body-mass index | 2.03 | 1.59 | 2.47 | 0 |
| Burkina Faso | Low physical activity | -0.23 | -0.36 | -0.1 | 0.001 |
| Marshall Islands | All cause | 1.09 | 0.88 | 1.3 | 0 |
| Marshall Islands | Tobacco | 0.95 | 0.71 | 1.19 | 0 |
| Marshall Islands | Alcohol use | 1.8 | 1.58 | 2.02 | 0 |
| Marshall Islands | High fasting plasma glucose | 2.03 | 1.68 | 2.37 | 0 |
| Marshall Islands | High body-mass index | 2.46 | 1.98 | 2.95 | 0 |
| Marshall Islands | Low physical activity | 1.08 | 0.89 | 1.27 | 0 |
| Colombia | All cause | -1.01 | -1.15 | -0.88 | 0 |
| Colombia | Tobacco | -2.5 | -2.66 | -2.35 | 0 |
| Colombia | Alcohol use | -1.25 | -1.44 | -1.06 | 0 |
| Colombia | High fasting plasma glucose | -1.67 | -1.92 | -1.43 | 0 |
| Colombia | High body-mass index | -0.43 | -0.58 | -0.27 | 0 |
| Colombia | Low physical activity | -1.04 | -1.17 | -0.9 | 0 |
| Libya | All cause | 1.18 | 0.95 | 1.41 | 0 |
| Libya | Tobacco | 1.68 | 1.45 | 1.91 | 0 |
| Libya | Alcohol use | -2.12 | -3.92 | -0.28 | 0.025 |
| Libya | High fasting plasma glucose | 2.19 | 1.9 | 2.47 | 0 |
| Libya | High body-mass index | 1.7 | 1.4 | 2 | 0 |
| Libya | Low physical activity | 1.23 | 1.01 | 1.46 | 0 |
| Singapore | All cause | -1.02 | -1.28 | -0.76 | 0 |
| Singapore | Tobacco | -1.39 | -1.66 | -1.13 | 0 |
| Singapore | Alcohol use | -1.16 | -1.46 | -0.85 | 0 |
| Singapore | High fasting plasma glucose | -1.57 | -1.98 | -1.15 | 0 |
| Singapore | High body-mass index | 1.51 | 0.8 | 2.22 | 0 |
| Singapore | Low physical activity | -1 | -1.21 | -0.79 | 0 |
| Federated States of Micron | All cause | 0.73 | 0.61 | 0.86 | 0 |
| Federated States of Micron | Tobacco | 0.6 | 0.49 | 0.71 | 0 |
| Federated States of Micron | Alcohol use | -0.16 | -0.41 | 0.08 | 0.188 |
| Federated States of Micron | High fasting plasma glucose | 1.78 | 1.54 | 2.02 | 0 |
| Federated States of Micron | High body-mass index | 1.3 | 0.89 | 1.72 | 0 |
| Federated States of Micron | Low physical activity | 0.67 | 0.56 | 0.78 | 0 |
| Swaziland | All cause | 1.39 | 0.91 | 1.88 | 0 |
| Swaziland | Tobacco | 0.85 | 0.4 | 1.3 | 0.001 |
| Swaziland | Alcohol use | 0.33 | -0.47 | 1.13 | 0.403 |
| Swaziland | High fasting plasma glucose | 2.41 | 1.83 | 3 | 0 |
| Swaziland | High body-mass index | 2.33 | 1.76 | 2.9 | 0 |
| Swaziland | Low physical activity | 1.41 | 0.94 | 1.89 | 0 |
| Grenada | All cause | 0.94 | 0.45 | 1.44 | 0.001 |
| Grenada | Tobacco | 0.73 | 0.28 | 1.18 | 0.002 |
| Grenada | Alcohol use | 0.32 | -0.21 | 0.85 | 0.225 |
| Grenada | High fasting plasma glucose | 1.61 | 1.08 | 2.14 | 0 |
| Grenada | High body-mass index | 2.8 | 2.51 | 3.08 | 0 |
| Grenada | Low physical activity | 1.01 | 0.52 | 1.51 | 0 |
| Jordan | All cause | -0.33 | -0.79 | 0.13 | 0.149 |
| Jordan | Tobacco | -0.51 | -1.01 | -0.01 | 0.046 |
| Jordan | Alcohol use | 2.22 | 1 | 3.45 | 0.001 |

| Jordan | High fasting plasma glucose | 0.63 | 0.08 | 1.19 | 0.027 |
| --- | --- | --- | --- | --- | --- |
| Jordan | High body-mass index | 3.54 | 2.89 | 4.19 | 0 |
| Jordan | Low physical activity | -0.11 | -0.54 | 0.31 | 0.588 |
| Switzerland | All cause | -2.11 | -2.22 | -1.99 | 0 |
| Switzerland | Tobacco | -2.47 | -2.58 | -2.35 | 0 |
| Switzerland | Alcohol use | -2.55 | -2.66 | -2.44 | 0 |
| Switzerland | High fasting plasma glucose | -1.6 | -1.76 | -1.44 | 0 |
| Switzerland | High body-mass index | -1.3 | -1.47 | -1.12 | 0 |
| Switzerland | Low physical activity | -2.03 | -2.15 | -1.9 | 0 |
| Zimbabwe | All cause | 2.64 | 1.8 | 3.49 | 0 |
| Zimbabwe | Tobacco | 2.99 | 2.12 | 3.85 | 0 |
| Zimbabwe | Alcohol use | 2.68 | 1.79 | 3.58 | 0 |
| Zimbabwe | High fasting plasma glucose | 4.15 | 3.22 | 5.08 | 0 |
| Zimbabwe | High body-mass index | 3.2 | 2.49 | 3.93 | 0 |
| Zimbabwe | Low physical activity | 2.63 | 1.79 | 3.48 | 0 |
| Nicaragua | All cause | 0.78 | 0.67 | 0.88 | 0 |
| Nicaragua | Tobacco | 0.43 | 0.33 | 0.53 | 0 |
| Nicaragua | Alcohol use | 0.36 | 0.14 | 0.59 | 0.002 |
| Nicaragua | High fasting plasma glucose | 1.07 | 0.96 | 1.17 | 0 |
| Nicaragua | High body-mass index | 2.9 | 2.59 | 3.21 | 0 |
| Nicaragua | Low physical activity | 0.95 | 0.84 | 1.06 | 0 |
| France | All cause | -1.33 | -1.54 | -1.12 | 0 |
| France | Tobacco | -1.48 | -1.74 | -1.21 | 0 |
| France | Alcohol use | -2.16 | -2.4 | -1.93 | 0 |
| France | High fasting plasma glucose | 0.09 | -0.1 | 0.28 | 0.354 |
| France | High body-mass index | -0.29 | -0.62 | 0.04 | 0.079 |
| France | Low physical activity | -1.28 | -1.48 | -1.08 | 0 |
| Guyana | All cause | 1.06 | 0.7 | 1.42 | 0 |
| Guyana | Tobacco | 0.49 | 0.09 | 0.9 | 0.018 |
| Guyana | Alcohol use | -0.7 | -1.16 | -0.24 | 0.005 |
| Guyana | High fasting plasma glucose | 1.62 | 1.14 | 2.1 | 0 |
| Guyana | High body-mass index | 1.78 | 1.3 | 2.27 | 0 |
| Guyana | Low physical activity | 1.06 | 0.7 | 1.42 | 0 |
| United Kingdom | All cause | -2.04 | -2.15 | -1.93 | 0 |
| United Kingdom | Tobacco | -3.6 | -3.66 | -3.54 | 0 |
| United Kingdom | Alcohol use | -2.18 | -2.32 | -2.04 | 0 |
| United Kingdom | High fasting plasma glucose | -0.54 | -0.75 | -0.33 | 0 |
| United Kingdom | High body-mass index | -1.34 | -1.45 | -1.23 | 0 |
| United Kingdom | Low physical activity | -1.95 | -2.05 | -1.84 | 0 |
| Germany | All cause | -1.27 | -1.39 | -1.14 | 0 |
| Germany | Tobacco | -2.12 | -2.42 | -1.82 | 0 |
| Germany | Alcohol use | -1.86 | -1.98 | -1.75 | 0 |
| Germany | High fasting plasma glucose | -0.42 | -0.74 | -0.1 | 0.011 |
| Germany | High body-mass index | 0.15 | 0.03 | 0.27 | 0.014 |
| Germany | Low physical activity | -1.1 | -1.22 | -0.97 | 0 |
| Norway | All cause | -1.68 | -1.85 | -1.51 | 0 |
| Norway | Tobacco | -2.52 | -2.9 | -2.14 | 0 |
| Norway | Alcohol use | -1.34 | -1.58 | -1.1 | 0 |
| Norway | High fasting plasma glucose | -0.87 | -1.01 | -0.73 | 0 |
| Norway | High body-mass index | -0.24 | -0.38 | -0.11 | 0.001 |
| Norway | Low physical activity | -1.19 | -1.34 | -1.04 | 0 |
| Portugal | All cause | -1.83 | -1.96 | -1.7 | 0 |
| Portugal | Tobacco | -2.2 | -2.36 | -2.04 | 0 |
| Portugal | Alcohol use | -2.41 | -2.54 | -2.28 | 0 |
| Portugal | High fasting plasma glucose | -0.97 | -1.17 | -0.78 | 0 |
| Portugal | High body-mass index | -0.26 | -0.58 | 0.05 | 0.098 |
| Portugal | Low physical activity | -1.76 | -1.89 | -1.62 | 0 |
| Israel | All cause | -1.83 | -2.13 | -1.53 | 0 |

| Israel | Tobacco | -2.9 | -3.22 | -2.58 | 0 |
| --- | --- | --- | --- | --- | --- |
| Israel | Alcohol use | -1.06 | -1.23 | -0.88 | 0 |
| Israel | High fasting plasma glucose | -1.07 | -1.67 | -0.47 | 0.001 |
| Israel | High body-mass index | -0.58 | -1.23 | 0.07 | 0.08 |
| Israel | Low physical activity | -1.75 | -2.06 | -1.44 | 0 |
| Italy | All cause | -1.43 | -1.51 | -1.35 | 0 |
| Italy | Tobacco | -1.98 | -2.25 | -1.71 | 0 |
| Italy | Alcohol use | -2.88 | -2.98 | -2.79 | 0 |
| Italy | High fasting plasma glucose | -0.87 | -0.99 | -0.76 | 0 |
| Italy | High body-mass index | -0.52 | -0.68 | -0.35 | 0 |
| Italy | Low physical activity | -1.38 | -1.46 | -1.3 | 0 |
| Austria | All cause | -1.53 | -1.66 | -1.4 | 0 |
| Austria | Tobacco | -1.47 | -1.58 | -1.36 | 0 |
| Austria | Alcohol use | -2.02 | -2.14 | -1.89 | 0 |
| Austria | High fasting plasma glucose | -0.07 | -0.21 | 0.07 | 0.31 |
| Austria | High body-mass index | -0.21 | -0.32 | -0.11 | 0 |
| Austria | Low physical activity | -1.39 | -1.52 | -1.27 | 0 |
| Andorra | All cause | -0.78 | -1 | -0.55 | 0 |
| Andorra | Tobacco | -0.7 | -0.89 | -0.5 | 0 |
| Andorra | Alcohol use | -1.77 | -2 | -1.54 | 0 |
| Andorra | High fasting plasma glucose | 0.32 | 0.04 | 0.61 | 0.026 |
| Andorra | High body-mass index | 0.39 | 0.19 | 0.59 | 0.001 |
| Andorra | Low physical activity | -0.68 | -0.9 | -0.46 | 0 |
| Chile | All cause | -0.71 | -0.81 | -0.61 | 0 |
| Chile | Tobacco | -1.15 | -1.24 | -1.06 | 0 |
| Chile | Alcohol use | -0.72 | -1 | -0.43 | 0 |
| Chile | High fasting plasma glucose | -0.06 | -0.18 | 0.07 | 0.387 |
| Chile | High body-mass index | 0.61 | 0.2 | 1.01 | 0.005 |
| Chile | Low physical activity | -0.64 | -0.73 | -0.54 | 0 |
| Kuwait | All cause | -1.13 | -1.7 | -0.56 | 0 |
| Kuwait | Tobacco | -0.94 | -1.55 | -0.32 | 0.004 |
| Kuwait | Alcohol use | -4.69 | -5.72 | -3.66 | 0 |
| Kuwait | High fasting plasma glucose | -0.63 | -1.35 | 0.09 | 0.083 |
| Kuwait | High body-mass index | 1.75 | 1.09 | 2.42 | 0 |
| Kuwait | Low physical activity | -1.04 | -1.61 | -0.47 | 0.001 |
| Canada | All cause | -2 | -2.15 | -1.86 | 0 |
| Canada | Tobacco | -3.45 | -3.59 | -3.31 | 0 |
| Canada | Alcohol use | -1.94 | -2.08 | -1.8 | 0 |
| Canada | High fasting plasma glucose | -2.85 | -3.07 | -2.63 | 0 |
| Canada | High body-mass index | -0.85 | -1.11 | -0.59 | 0 |
| Canada | Low physical activity | -1.95 | -2.11 | -1.79 | 0 |
| Trinidad and Tobago | All cause | -0.63 | -0.82 | -0.45 | 0 |
| Trinidad and Tobago | Tobacco | -0.55 | -0.73 | -0.36 | 0 |
| Trinidad and Tobago | Alcohol use | -0.5 | -0.81 | -0.19 | 0.003 |
| Trinidad and Tobago | High fasting plasma glucose | -0.66 | -0.85 | -0.47 | 0 |
| Trinidad and Tobago | High body-mass index | 1.08 | 0.85 | 1.3 | 0 |
| Trinidad and Tobago | Low physical activity | -0.62 | -0.81 | -0.44 | 0 |
| Spain | All cause | -1.82 | -1.89 | -1.74 | 0 |
| Spain | Tobacco | -1.61 | -1.8 | -1.43 | 0 |
| Spain | Alcohol use | -2.33 | -2.4 | -2.26 | 0 |
| Spain | High fasting plasma glucose | -1.31 | -1.45 | -1.17 | 0 |
| Spain | High body-mass index | -0.89 | -1.15 | -0.63 | 0 |
| Spain | Low physical activity | -1.7 | -1.79 | -1.62 | 0 |
| Sweden | All cause | -0.97 | -1.05 | -0.9 | 0 |
| Sweden | Tobacco | -1.45 | -1.54 | -1.35 | 0 |
| Sweden | Alcohol use | -1.08 | -1.24 | -0.92 | 0 |
| Sweden | High fasting plasma glucose | 0.32 | 0.23 | 0.4 | 0 |
| Sweden | High body-mass index | 0.24 | 0.09 | 0.39 | 0.002 |

| Sweden | Low physical activity | -0.9 | -0.98 | -0.83 | 0 |
| --- | --- | --- | --- | --- | --- |
| Belgium | All cause | -1.74 | -1.86 | -1.63 | 0 |
| Belgium | Tobacco | -2.11 | -2.36 | -1.87 | 0 |
| Belgium | Alcohol use | -1.38 | -1.55 | -1.2 | 0 |
| Belgium | High fasting plasma glucose | -0.97 | -1.09 | -0.85 | 0 |
| Belgium | High body-mass index | -0.54 | -0.71 | -0.37 | 0 |
| Belgium | Low physical activity | -1.67 | -1.79 | -1.56 | 0 |
| Finland | All cause | -1.05 | -1.19 | -0.9 | 0 |
| Finland | Tobacco | -2.52 | -2.67 | -2.36 | 0 |
| Finland | Alcohol use | -1.11 | -1.33 | -0.89 | 0 |
| Finland | High fasting plasma glucose | -0.06 | -0.23 | 0.1 | 0.423 |
| Finland | High body-mass index | 0.81 | 0.58 | 1.03 | 0 |
| Finland | Low physical activity | -0.89 | -1.03 | -0.75 | 0 |
| Sudan | All cause | 0.44 | 0.39 | 0.5 | 0 |
| Sudan | Tobacco | -0.03 | -0.19 | 0.13 | 0.742 |
| Sudan | Alcohol use | 0.46 | 0.39 | 0.53 | 0 |
| Sudan | High fasting plasma glucose | 1.5 | 1.37 | 1.63 | 0 |
| Sudan | High body-mass index | 3.94 | 3.77 | 4.1 | 0 |
| Sudan | Low physical activity | 0.41 | 0.26 | 0.55 | 0 |
| Denmark | All cause | -1.96 | -2.15 | -1.77 | 0 |
| Denmark | Tobacco | -3.34 | -3.65 | -3.03 | 0 |
| Denmark | Alcohol use | -2.23 | -2.58 | -1.89 | 0 |
| Denmark | High fasting plasma glucose | -0.4 | -0.69 | -0.11 | 0.008 |
| Denmark | High body-mass index | -0.41 | -0.68 | -0.14 | 0.004 |
| Denmark | Low physical activity | -1.78 | -1.98 | -1.59 | 0 |
| Uruguay | All cause | -0.88 | -0.99 | -0.78 | 0 |
| Uruguay | Tobacco | -1.19 | -1.28 | -1.1 | 0 |
| Uruguay | Alcohol use | -1.52 | -1.75 | -1.28 | 0 |
| Uruguay | High fasting plasma glucose | -0.83 | -0.97 | -0.69 | 0 |
| Uruguay | High body-mass index | 0.98 | 0.56 | 1.41 | 0 |
| Uruguay | Low physical activity | -0.78 | -0.89 | -0.67 | 0 |
| Argentina | All cause | -0.49 | -0.66 | -0.32 | 0 |
| Argentina | Tobacco | -1.04 | -1.23 | -0.84 | 0 |
| Argentina | Alcohol use | -1.58 | -1.7 | -1.45 | 0 |
| Argentina | High fasting plasma glucose | 0.14 | -0.08 | 0.36 | 0.215 |
| Argentina | High body-mass index | 1.7 | 1.21 | 2.19 | 0 |
| Argentina | Low physical activity | -0.4 | -0.56 | -0.23 | 0 |
| Low-middle SDI | All cause | 0.48 | 0.37 | 0.58 | 0 |
| Low-middle SDI | Tobacco | -0.18 | -0.36 | 0.01 | 0.058 |
| Low-middle SDI | Alcohol use | 0.11 | -0.1 | 0.31 | 0.297 |
| Low-middle SDI | High fasting plasma glucose | 1.39 | 1.29 | 1.5 | 0 |
| Low-middle SDI | High body-mass index | 2.95 | 2.87 | 3.03 | 0 |
| Low-middle SDI | Low physical activity | 0.55 | 0.45 | 0.65 | 0 |
| Low SDI | All cause | 0.57 | 0.37 | 0.77 | 0 |
| Low SDI | Tobacco | 0.42 | 0.24 | 0.6 | 0 |
| Low SDI | Alcohol use | -0.05 | -0.2 | 0.09 | 0.47 |
| Low SDI | High fasting plasma glucose | 1.42 | 1.27 | 1.57 | 0 |
| Low SDI | High body-mass index | 2.88 | 2.7 | 3.06 | 0 |
| Low SDI | Low physical activity | 0.6 | 0.4 | 0.8 | 0 |
| Tropical Latin America | All cause | -0.34 | -0.53 | -0.14 | 0.002 |
| Tropical Latin America | Tobacco | -1.64 | -2.02 | -1.27 | 0 |
| Tropical Latin America | Alcohol use | 0.05 | -0.37 | 0.48 | 0.796 |
| Tropical Latin America | High fasting plasma glucose | -0.7 | -0.98 | -0.43 | 0 |
| Tropical Latin America | High body-mass index | 1.33 | 0.97 | 1.69 | 0 |
| Tropical Latin America | Low physical activity | -0.33 | -0.53 | -0.14 | 0.002 |
| Central Latin America | All cause | 0.01 | -0.06 | 0.09 | 0.7 |
| Central Latin America | Tobacco | -1.46 | -1.57 | -1.34 | 0 |
| Central Latin America | Alcohol use | -0.58 | -0.67 | -0.5 | 0 |

| Central Latin America | High fasting plasma glucose | 0.22 | 0.06 | 0.38 | 0.01 |
| --- | --- | --- | --- | --- | --- |
| Central Latin America | High body-mass index | 1.45 | 1.36 | 1.53 | 0 |
| Central Latin America | Low physical activity | -0.03 | -0.11 | 0.06 | 0.523 |
| East Asia | All cause | -0.03 | -0.16 | 0.11 | 0.666 |
| East Asia | Tobacco | -0.49 | -0.61 | -0.37 | 0 |
| East Asia | Alcohol use | 0.51 | 0.23 | 0.8 | 0.001 |
| East Asia | High fasting plasma glucose | 0.98 | 0.74 | 1.23 | 0 |
| East Asia | High body-mass index | 3.63 | 3.46 | 3.81 | 0 |
| East Asia | Low physical activity | 0.23 | 0.09 | 0.37 | 0.002 |
| Andean Latin America | All cause | 0.18 | 0.04 | 0.33 | 0.013 |
| Andean Latin America | Tobacco | -0.05 | -0.21 | 0.11 | 0.531 |
| Andean Latin America | Alcohol use | -0.46 | -0.63 | -0.3 | 0 |
| Andean Latin America | High fasting plasma glucose | 0.99 | 0.9 | 1.09 | 0 |
| Andean Latin America | High body-mass index | 3.2 | 3.01 | 3.4 | 0 |
| Andean Latin America | Low physical activity | 0.27 | 0.13 | 0.41 | 0.001 |
| Australasia | All cause | -1.57 | -1.68 | -1.45 | 0 |
| Australasia | Tobacco | -3.02 | -3.21 | -2.84 | 0 |
| Australasia | Alcohol use | -1.87 | -2.06 | -1.69 | 0 |
| Australasia | High fasting plasma glucose | -1.46 | -1.67 | -1.25 | 0 |
| Australasia | High body-mass index | -0.04 | -0.19 | 0.1 | 0.552 |
| Australasia | Low physical activity | -1.46 | -1.57 | -1.36 | 0 |
| Caribbean | All cause | 0.32 | 0.25 | 0.4 | 0 |
| Caribbean | Tobacco | -0.25 | -0.32 | -0.19 | 0 |
| Caribbean | Alcohol use | 0.76 | 0.64 | 0.88 | 0 |
| Caribbean | High fasting plasma glucose | 0.57 | 0.49 | 0.64 | 0 |
| Caribbean | High body-mass index | 1.72 | 1.56 | 1.87 | 0 |
| Caribbean | Low physical activity | 0.28 | 0.21 | 0.36 | 0 |
| Eastern Europe | All cause | -0.29 | -0.7 | 0.12 | 0.162 |
| Eastern Europe | Tobacco | -0.64 | -0.99 | -0.28 | 0.001 |
| Eastern Europe | Alcohol use | 0.48 | 0 | 0.97 | 0.051 |
| Eastern Europe | High fasting plasma glucose | 0.33 | -0.07 | 0.74 | 0.105 |
| Eastern Europe | High body-mass index | 2.21 | 1.67 | 2.76 | 0 |
| Eastern Europe | Low physical activity | -0.06 | -0.41 | 0.29 | 0.739 |
| High-income North America | All cause | -1.79 | -1.91 | -1.68 | 0 |
| High-income North America | Tobacco | -3.29 | -3.39 | -3.2 | 0 |
| High-income North America | Alcohol use | -1.65 | -1.76 | -1.55 | 0 |
| High-income North America | High fasting plasma glucose | 0.08 | -0.22 | 0.38 | 0.582 |
| High-income North America | High body-mass index | -0.68 | -0.78 | -0.58 | 0 |
| High-income North America | Low physical activity | -1.85 | -1.97 | -1.73 | 0 |
| Central Asia | All cause | 0.32 | 0.14 | 0.5 | 0.001 |
| Central Asia | Tobacco | 0.05 | -0.11 | 0.2 | 0.537 |
| Central Asia | Alcohol use | 0.38 | 0.06 | 0.71 | 0.021 |
| Central Asia | High fasting plasma glucose | 1.42 | 1.16 | 1.67 | 0 |
| Central Asia | High body-mass index | 2.17 | 1.82 | 2.52 | 0 |
| Central Asia | Low physical activity | 0.51 | 0.33 | 0.7 | 0 |
| High-income Asia Pacific | All cause | 0.8 | 0.57 | 1.03 | 0 |
| High-income Asia Pacific | Tobacco | -0.31 | -0.53 | -0.09 | 0.007 |
| High-income Asia Pacific | Alcohol use | 0.46 | 0.24 | 0.69 | 0 |
| High-income Asia Pacific | High fasting plasma glucose | 1.34 | 1.13 | 1.55 | 0 |
| High-income Asia Pacific | High body-mass index | 2.67 | 2.33 | 3.02 | 0 |
| High-income Asia Pacific | Low physical activity | 0.79 | 0.59 | 0.98 | 0 |
| Central Europe | All cause | -0.36 | -0.46 | -0.25 | 0 |
| Central Europe | Tobacco | -0.79 | -0.9 | -0.67 | 0 |
| Central Europe | Alcohol use | -0.66 | -0.74 | -0.57 | 0 |
| Central Europe | High fasting plasma glucose | 0.43 | 0.35 | 0.51 | 0 |
| Central Europe | High body-mass index | 1.22 | 1.03 | 1.42 | 0 |
| Central Europe | Low physical activity | -0.14 | -0.24 | -0.04 | 0.007 |
| North Africa and Middle Ea | All cause | 0.25 | 0.06 | 0.45 | 0.013 |

| North Africa and Middle Ea | Tobacco | -0.21 | -0.43 | 0.02 | 0.067 |
| --- | --- | --- | --- | --- | --- |
| North Africa and Middle Ea | Alcohol use | -1.18 | -1.56 | -0.81 | 0 |
| North Africa and Middle Ea | High fasting plasma glucose | 1.14 | 1.01 | 1.26 | 0 |
| North Africa and Middle Ea | High body-mass index | 1.96 | 1.74 | 2.19 | 0 |
| North Africa and Middle Ea | Low physical activity | 0.38 | 0.21 | 0.55 | 0 |
| Southeast Asia | All cause | 0.01 | -0.09 | 0.12 | 0.782 |
| Southeast Asia | Tobacco | -0.14 | -0.31 | 0.03 | 0.096 |
| Southeast Asia | Alcohol use | 1.87 | 1.62 | 2.13 | 0 |
| Southeast Asia | High fasting plasma glucose | 0.99 | 0.95 | 1.03 | 0 |
| Southeast Asia | High body-mass index | 3.42 | 3.27 | 3.56 | 0 |
| Southeast Asia | Low physical activity | 0.04 | -0.07 | 0.15 | 0.457 |
| Western Sub-Saharan Afric | All cause | 0.21 | 0.13 | 0.28 | 0 |
| Western Sub-Saharan Afric | Tobacco | -0.66 | -0.75 | -0.57 | 0 |
| Western Sub-Saharan Afric | Alcohol use | -0.64 | -0.88 | -0.39 | 0 |
| Western Sub-Saharan Afric | High fasting plasma glucose | 1.36 | 1.27 | 1.45 | 0 |
| Western Sub-Saharan Afric | High body-mass index | 0.79 | 0.5 | 1.07 | 0 |
| Western Sub-Saharan Afric | Low physical activity | 0.28 | 0.21 | 0.36 | 0 |
| South Asia | All cause | 0.85 | 0.67 | 1.03 | 0 |
| South Asia | Tobacco | 0.05 | -0.15 | 0.24 | 0.634 |
| South Asia | Alcohol use | 1.55 | 1.34 | 1.76 | 0 |
| South Asia | High fasting plasma glucose | 2.11 | 1.99 | 2.24 | 0 |
| South Asia | High body-mass index | 6 | 5.56 | 6.44 | 0 |
| South Asia | Low physical activity | 0.89 | 0.71 | 1.07 | 0 |
| Southern Sub-Saharan Afri | All cause | 0.6 | 0.08 | 1.12 | 0.024 |
| Southern Sub-Saharan Afri | Tobacco | -0.76 | -1.22 | -0.31 | 0.002 |
| Southern Sub-Saharan Afri | Alcohol use | 0.43 | -0.1 | 0.96 | 0.109 |
| Southern Sub-Saharan Afri | High fasting plasma glucose | 1.92 | 1.41 | 2.44 | 0 |
| Southern Sub-Saharan Afri | High body-mass index | 3.07 | 2.67 | 3.47 | 0 |
| Southern Sub-Saharan Afri | Low physical activity | 0.59 | 0.14 | 1.05 | 0.013 |
| Southern Latin America | All cause | -0.69 | -0.82 | -0.55 | 0 |
| Southern Latin America | Tobacco | -1.21 | -1.37 | -1.06 | 0 |
| Southern Latin America | Alcohol use | -1.57 | -1.68 | -1.45 | 0 |
| Southern Latin America | High fasting plasma glucose | -0.15 | -0.34 | 0.04 | 0.113 |
| Southern Latin America | High body-mass index | 1.3 | 0.82 | 1.78 | 0 |
| Southern Latin America | Low physical activity | -0.6 | -0.72 | -0.47 | 0 |
| Eastern Sub-Saharan Afric | All cause | -0.43 | -0.6 | -0.27 | 0 |
| Eastern Sub-Saharan Afric | Tobacco | -0.34 | -0.49 | -0.19 | 0 |
| Eastern Sub-Saharan Afric | Alcohol use | -0.48 | -0.61 | -0.34 | 0 |
| Eastern Sub-Saharan Afric | High fasting plasma glucose | 0.07 | -0.07 | 0.21 | 0.293 |
| Eastern Sub-Saharan Afric | High body-mass index | 3.07 | 2.86 | 3.29 | 0 |
| Eastern Sub-Saharan Afric | Low physical activity | -0.54 | -0.71 | -0.36 | 0 |
| Oceania | All cause | 0.46 | 0.38 | 0.53 | 0 |
| Oceania | Tobacco | 0.38 | 0.31 | 0.44 | 0 |
| Oceania | Alcohol use | 0.58 | 0.48 | 0.68 | 0 |
| Oceania | High fasting plasma glucose | 1.16 | 1.12 | 1.21 | 0 |
| Oceania | High body-mass index | 1.07 | 0.89 | 1.26 | 0 |
| Oceania | Low physical activity | 0.47 | 0.39 | 0.55 | 0 |
| Western Europe | All cause | -1.55 | -1.61 | -1.49 | 0 |
| Western Europe | Tobacco | -2.32 | -2.42 | -2.22 | 0 |
| Western Europe | Alcohol use | -2.13 | -2.2 | -2.05 | 0 |
| Western Europe | High fasting plasma glucose | -0.59 | -0.67 | -0.51 | 0 |
| Western Europe | High body-mass index | -0.5 | -0.65 | -0.36 | 0 |
| Western Europe | Low physical activity | -1.45 | -1.51 | -1.39 | 0 |
| Central Sub-Saharan Africa | All cause | 0.51 | 0.43 | 0.6 | 0 |
| Central Sub-Saharan Africa | Tobacco | 0.47 | 0.42 | 0.52 | 0 |
| Central Sub-Saharan Africa | Alcohol use | 1.27 | 1 | 1.54 | 0 |
| Central Sub-Saharan Africa | High fasting plasma glucose | 1.22 | 1.11 | 1.32 | 0 |
| Central Sub-Saharan Africa | High body-mass index | 1.27 | 1 | 1.54 | 0 |

| Central Sub-Saharan Africa | Low physical activity | 0.55 | 0.45 | 0.65 | 0 |
| --- | --- | --- | --- | --- | --- |
